# Azithromycin alters spatial and temporal dynamics of airway microbiota in idiopathic pulmonary fibrosis

**DOI:** 10.1183/23120541.00720-2022

**Published:** 2023-05-22

**Authors:** Pieter-Jan Gijs, Cécile Daccord, Eric Bernasconi, Martin Brutsche, Christian F. Clarenbach, Katrin Hostettler, Sabina A. Guler, Louis Mercier, Niki Ubags, Manuela Funke-Chambour, Christophe von Garnier

**Affiliations:** 1 Division of Pulmonology, Department of Medicine, CHUV, Lausanne University Hospital, University of Lausanne, Lausanne, Switzerland; 2 Lungenzentrum, Kantonsspital St Gallen, St Gallen, Switzerland; 3 Division of Pulmonary Medicine, University Hospital of Zurich, Zurich, Switzerland; 4 Clinics of Respiratory Medicine, University Hospital Basel, Basel, Switzerland; 5 Department of Pulmonary Medicine, Inselspital, Bern University Hospital, Bern, Switzerland; 6 Joint first authors; 7 Joint senior authors

## Abstract

**Background:**

High bacterial burden in the lung microbiota predicts progression of idiopathic pulmonary fibrosis (IPF). Azithromycin (AZT) is a macrolide antibiotic known to alter the lung microbiota in several chronic pulmonary diseases, and observational studies have shown a positive effect of AZT on mortality and hospitalisation rate in IPF. However, the effect of AZT on the lung microbiota in IPF remains unknown.

**Methods:**

We sought to determine the impact of a 3-month course of AZT on the lung microbiota in IPF. We assessed sputum and oropharyngeal swab specimens from 24 adults with IPF included in a randomised controlled crossover trial of oral AZT 500 mg 3 times per week. 16S rRNA gene amplicon sequencing and quantitative PCR (qPCR) were performed to assess bacterial communities. Antibiotic resistance genes (ARGs) were assessed using real-time qPCR.

**Results:**

AZT significantly decreased community diversity with a stronger and more persistent effect in the lower airways (sputum). AZT treatment altered the temporal kinetics of the upper (oropharyngeal swab) and lower airway microbiota, increasing community similarity between the two sites for 1 month after macrolide cessation. Patients with an increase in ARG carriage had lower bacterial density and enrichment of the genus *Streptococcus*. In contrast, patients with more stable ARG carriage had higher bacterial density and enrichment in *Prevotella*.

**Conclusions:**

AZT caused sustained changes in the diversity and composition of the upper and lower airway microbiota in IPF, with effects on the temporal and spatial dynamics between the two sites.

## Abstract 2

**In IPF, azithromycin alters the dynamics of the microbiota between the upper and lower airways and community turnover over time, with a decrease in richness without impacting the bacterial density.** https://bit.ly/3Jp9xOg

## Introduction

Idiopathic pulmonary fibrosis (IPF) is a progressive and fatal interstitial lung disease of unknown origin, and is the most common and severe form of the idiopathic interstitial pneumonias [1, 2].

In healthy subjects, the composition of the microbiota in the upper respiratory tract (URT) and lower respiratory tract (LRT) has considerable similarities [3]. In the LRT, the influx of bacteria by microaspiration is counterbalanced by mucociliary clearance, resulting in a physiological turnover. Respiratory health is therefore associated with a dynamic turnover of the microbiota between the URT and LRT.

Although alterations in the microbiota have not been found in lung tissue in end-stage IPF [4], converging evidence suggests that the spatial and temporal dynamics of the airway microbiota are disturbed during the disease course. In IPF, the LRT microbiota is more abundant [5, 6] and less diverse [7], suggesting either greater local growth, accumulation due to impaired clearance or both. One consequence is greater dissimilarity between the URT and LRT microbiota, with the LRT microbiota carrying a majority of genes absent in the URT [8]. This, alongside evidence from longitudinal studies on persistence of LRT microbiota disturbances [9], provides additional evidence for a lower microbiota turnover in IPF.

Pre-clinical data suggest that disruption of the LRT microbiota precedes chronic lung epithelial injury and repair [7, 10], potentially perpetuating inflammation [9]. Bacterial load is currently the LRT microbiota feature most consistently associated with disease, in terms of progression, exacerbations and mortality. Bacterial load is higher in IPF patients than in healthy subjects, making it a potential therapeutic target [11]. Although antimicrobial treatment with doxycycline or co-trimoxazole in addition to standard care did not improve time to death or nonelective hospitalisation [12], the effects of microbiome alterations by disease or drug treatment are poorly understood.

A single-centre retrospective study showed good tolerance to prophylactic azithromycin (AZT) in IPF patients, with fewer nonelective hospitalisations [13]. Also, treatment with AZT during acute IPF exacerbations improved survival rates compared with fluoroquinolones in a retrospective single-centre study [14].

The impact of AZT on the respiratory microbiota in IPF is unknown. In patients with severe asthma and emphysema, multiple effects on the composition and structure of the LRT microbiota as well as increased anti-inflammatory bacterial metabolites have been reported [15, 16]. This effect of AZT on the LRT microbiota was independent of a decrease in bacterial load.

Acquired macrolide resistance is a global health concern and macrolide administration increases the carriage of macrolide-resistant bacteria in the URT [17, 18] and LRT [19]. Higher carriage was observed after AZT for seven genes involved in antibiotic resistance, five of which associated with macrolide resistance and two with tetracycline resistance [15]. Additionally, the airway resistome positively correlates with bacterial load in COPD [20].

The aim of this study is to explore the impact of a 3-month course of AZT on the airway microbiota of IPF patients.

## Materials and methods

### Study population and sample collection

This study is a *post hoc* analysis of samples collected during the study conducted by Guler *et al*. [21] (ClinicalTrials.gov: NCT02173145). The previous study was a multicentre, double-blind, randomised, placebo-controlled crossover trial to determine the effect of AZT on chronic cough in IPF patients (see supplementary material for details).

Patients underwent a 12-week treatment period with oral AZT 500 mg 3 times per week and a 12-week treatment period with placebo 3 times per week in randomised order. The two periods were separated by a 4-week washout period.

Sputum samples, either expectorated or induced by inhalation of a 3% sodium chloride solution, and oropharyngeal swab (OPS) samples were collected both before and after AZT and placebo periods, and an additional specimen was collected after the 4-week washout period following the second treatment period. Samples were stored at −80°C until analysis. The study design and detailed sample collection are presented in supplementary figure E1 (see supplementary material for details of study population and sample collection).

### DNA extraction and 16S rRNA gene amplicon quantification

Sputum and OPS samples were treated with dithiothreitol to homogenise the mucosal phase and DNA was extracted using the DNeasy UltraClean Microbial Kit (Qiagen, Hilden, Germany), with inclusion of a lysozyme digestion step (see details in supplementary material).

Negative controls (n=11) underwent the same procedure and included blank swabs, blank sputum collection tubes as well as blank DNA extractions (reagent control).

To obtain a proxy of bacterial density, we determined the copy numbers of the 16S rRNA gene by quantitative PCR (qPCR) using previously reported primers specific to panbacteria (see supplementary table E1 for full sequences) [22]. Standard curves were obtained using purified amplicon products.

### Bacterial 16S rRNA gene amplicon sequencing

Amplicon sequencing targeted the V1–V2 region of the 16S rRNA gene with primers F-27 and R-338 (see supplementary table E1 for full sequences and supplementary material for details). Amplification was performed using the AccuPrime Taq DNA Polymerase High Fidelity Kit (Invitrogen, Waltham, MA, USA). No-template PCR reaction controls (n=2) were included. Libraries were loaded onto an Illumina MiSeq using pairwise chemistry, generating 2×250 bp read lengths (Lausanne Genomic Technologies Facility, University of Lausanne, Lausanne, Switzerland).

### Analysis of antibiotic resistance gene carriage

Quantification of antibiotic resistance gene (ARG) carriage targeting 23S ribosomal RNA methyltransferases (*erm*(B) and *erm*(F)), ATP-binding cassette ribosomal protection protein (*mel* and *msr*(E)), major facilitator superfamily antibiotic efflux pump (*mef*) and tetracycline-resistant ribosomal protection proteins (*tet*(M) and *tet*(W)) was performed on sputum specimens using dye-based (SsoAdvanced Universal SYBR Green; Bio-Rad, Hercules, CA, USA) or probe-based real-time PCR assays, using primer pairs, probes and conditions previously described [15]. The copy numbers of resistance genes per sample were normalised relative to the copy numbers of the 16S rRNA gene. To obtain a synthetic picture of ARG carriage per sample, the counts obtained for each individual gene were scaled from 0 to 1 to provide equal importance to each gene and the cumulative counts were reported.

### Bioinformatics and statistical analysis

All analyses were performed in R version 4.1.0 [23]. Demultiplexing, removal of chimeric and short reads, single-base resolution of reads into amplicon sequence variants (ASVs) using the Divisive Amplicon Denoising Algorithm 2 (DADA2) algorithm [24], and taxonomic annotation using the SILVA database [25] were performed using a dedicated pipeline available at https://github.com/chuvpne/dada2-pipeline. Contaminant screening was performed using the decontam package (supplementary figure E2). Subsequent analyses were performed on a rarefied dataset at a sequencing depth of 10 055 (supplementary figures E3–E5; see supplementary material for details on processing and quality control).

Multiple group comparisons were made using the Kruskal–Wallis test with Dunn's *post hoc* test and Holm's adjustment. The Wilcoxon signed-rank test was used to compare paired data. In all tests, we considered an α significance level of 0.05.

Comparisons of proportions of ASVs represented only in one condition or shared between two conditions were assessed using the Chi-squared goodness-of-fit test.

To test for differences in microbiota composition between the different phases of AZT treatment, we used a matrix built on either unweighted or weighted UniFrac distance and conducted a permutational multivariate ANOVA (PERMANOVA) with 999 permutations.

R scripts are available at https://github.com/CHUVpulmonology/Airway_microbiota-Lung_fibrosis-Azithromycin. The original sequencing data and the starting data for the analyses in R are available at https://zenodo.org/record/7065053#.ZAI92x_P0mA.

## Results

### Study cohort

Of the 25 patients who completed randomisation, 12 were initially randomised to intervention and 13 to placebo. 20 patients completed the study. The CONSORT (Consolidated Standards of Reporting Trials) diagram is shown in supplementary figure E6.

24 patients had at least one specimen, with a total of 67 sputum specimens and 90 OPS specimens. Table 1 shows patient demographics and clinical characteristics.

**TABLE 1:** Demographics and clinical characteristics of the study cohort (n=25) [21]

**Age, years**	67±8
**Male**	23 (92)
**Smoking status**	
Ex-smoker	17 (68)
Current smoker	1 (4)
**Diagnosis**	
Definite UIP pattern on HRCT	18 (72)
Surgical lung biopsy available	12 (48)
MDT discussion performed	19 (76)
**Treatment**	
Pirfenidone	9 (36)
Nintedanib	11 (44)
Proton pump inhibitor	13 (52)
Oxygen therapy	9 (36)
**Pulmonary function tests**	
TLC, % pred	60±12
FVC, % pred	65±16
FEV_1_/FVC, %	86±7
*D*_LCO_, % pred	43±16
**Comorbidities**	
Chronic rhinitis	8 (32)
Sinusitis	2 (8)
Gastro-oesophageal reflux disease	4 (16)
Cardiac disease	11 (44)
Pulmonary hypertension	3 (12)
Diabetes	4 (16)

Data are presented as n (%) or mean±sd. UIP: usual interstitial pneumonia; HRCT: high-resolution computed tomography; MDT: multidisciplinary team; TLC: total lung capacity; FVC: forced vital capacity; FEV_1_: forced expiratory volume in 1 s; *D*_LCO_: diffusing capacity of the lung for carbon monoxide.

Bacterial quantification, 16S rRNA gene amplicon sequencing and ARG analysis were performed on all specimens, except on one OPS specimen with insufficient DNA. 10 OPS specimens not meeting the read threshold of 10 055 were excluded from further sequencing analysis (supplementary figure E3). Finally, 67 sputum specimens and 79 OPS specimens were analysed.

To investigate temporal changes in respiratory microbiota linked to AZT, we distinguished five treatment phases: “PreAZT” for specimens collected before the start of AZT treatment, available only in patients who started with placebo (n=35: sputum=14; OPS=21), “StartAZT” for specimens collected at the beginning of AZT (n=31: sputum=15; OPS=16), “EndAZT” for specimens collected at the end of AZT (n=23: sputum=12; OPS=11), “PostAZT_1mo” for specimens collected 1 month after the end of treatment (n=32: sputum=14; OPS=18) and “PostAZT>=3mo” for specimens collected ≥3 months after the end of AZT (n=25: sputum=12; OPS=13).

### The respiratory microbiota of IPF patients is distinct from environmental noise

As our study used low-volume specimens from body sites with low microbial biomass, it was vulnerable to environmental noise [26]. However, we detected a significantly higher bacterial density (median (interquartile range (IQR)) 16S rRNA gene copies·µL^−1^ sputum) in both LRT (2×10^5^ (5.4×10^4^–4.5×10^5^); p<0.001) (supplementary figure E7a) and URT (1.7×10^5^ (6.5×10^4^–7.8×10^5^); p<0.001) (supplementary figure E7b) specimens compared with procedural controls (4×10^4^ (1.4×10^4^–5.6×10^4^)).

Rank abundance analysis further showed that the dominant bacteria were mostly different in patient samples *versus* controls (supplementary figure E7c).

### The upper and lower airway microbiota of IPF patients is altered after AZT treatment

**FIGURE 1:**
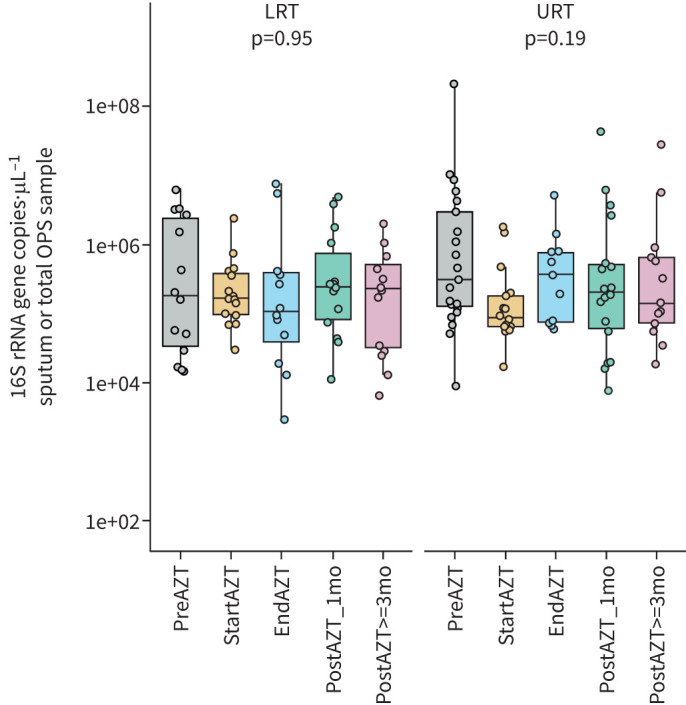
Quantitative PCR determination of 16S rRNA gene copy number per microlitre of sputum or total oropharyngeal swab (OPS) samples showing no significant change in bacterial density during the different phases of the study. Middle lines, boxes and whiskers represent the median, interquartile range (IQR) and 1.5×IQR, respectively. Dots represent samples. LRT: lower respiratory tract; URT: upper respiratory tract; AZT: azithromycin; mo: months. Kruskal–Wallis with Dunn's *post hoc* test.

**FIGURE 2:**
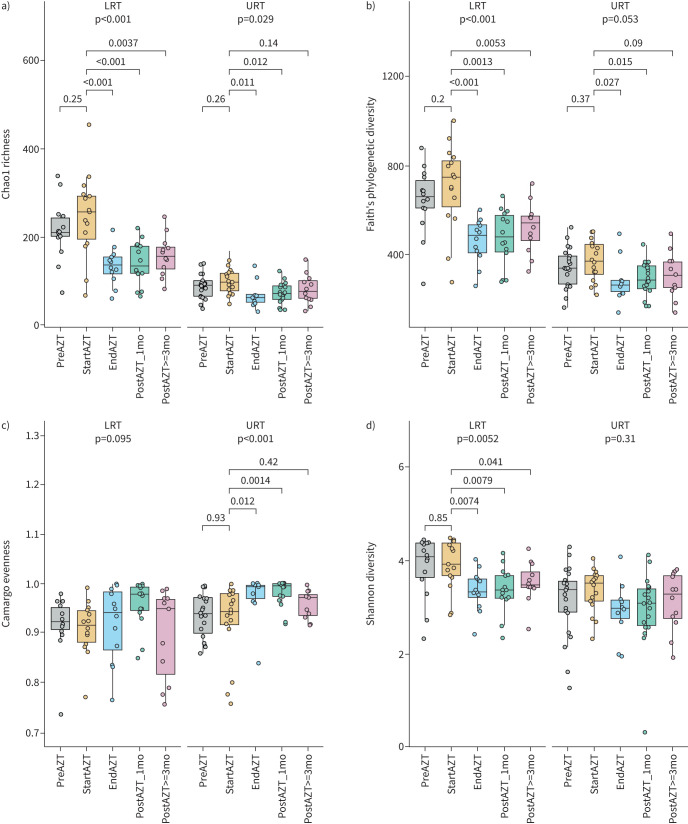
Decrease in community diversity after azithromycin (AZT) treatment. a–d) α diversity metrics showing a decrease in a) Chao1 richness and b) Faith's phylogenetic diversity during AZT treatment in the lower respiratory tract (LRT) and upper respiratory tract (URT), c) an increase in Camargo evenness in the URT, and d) a decrease in Shannon diversity in the LRT. Middle lines, boxes and whiskers represent the median, interquartile range (IQR) and 1.5×IQR, respectively. Dots represent samples. mo: months. Kruskal–Wallis with Dunn's *post hoc* test.

We found no difference in bacterial density between the different treatment phases in either the LRT (p=0.95) or URT (p=0.19) (figure 1), but we observed a decrease in community richness after AZT, with a stronger and more persistent effect in the LRT (p≤0.001) compared with the URT (p=0.029) (figure 2a). A decrease in bacterial phylogenetic diversity was also observed in the LRT (p≤0.001) and at the limit of significance in the URT (p=0.053), without reverting to the pre-treatment level until the end of the observation period for the LRT and more transiently for the URT (figure 2b). This decrease in α diversity between the start and end of AZT treatment, and the overall kinetics throughout the study, were confirmed by intra-individual (supplementary figure E8) and genus-level (supplementary figure E9) analyses.

In contrast, evenness increased transiently after treatment in the URT only (p<0.001) (figure 2c), while the Shannon diversity index reflected the effects of AZT on richness (figure 2d).

We observed no overall variation in the dominance of core community ASVs (*i.e.* abundance >0.1% in 50% of samples) in both the LRT (p=0.29) and URT (p=0.99) (supplementary figure E10), suggesting that the impact of AZT treatment did not primarily target the most abundant and prevalent bacteria.

Principal coordinate analysis (PCoA) based on unweighted UniFrac distance, which accounts for phylogeny, further supported variations in the microbiota present before and at the start of treatment compared with that found at the end and after treatment, with marked changes in the LRT (p=0.006) (supplementary figure E11a) but at the limit of significance in the URT (p=0.053) (supplementary figure E11b).

Together, these results show a broad impact of AZT on the respiratory microbiota of IPF patients.

### AZT treatment reduces spatial dissimilarity between the URT and LRT microbiota and affects their temporal dynamics

**FIGURE 3:**
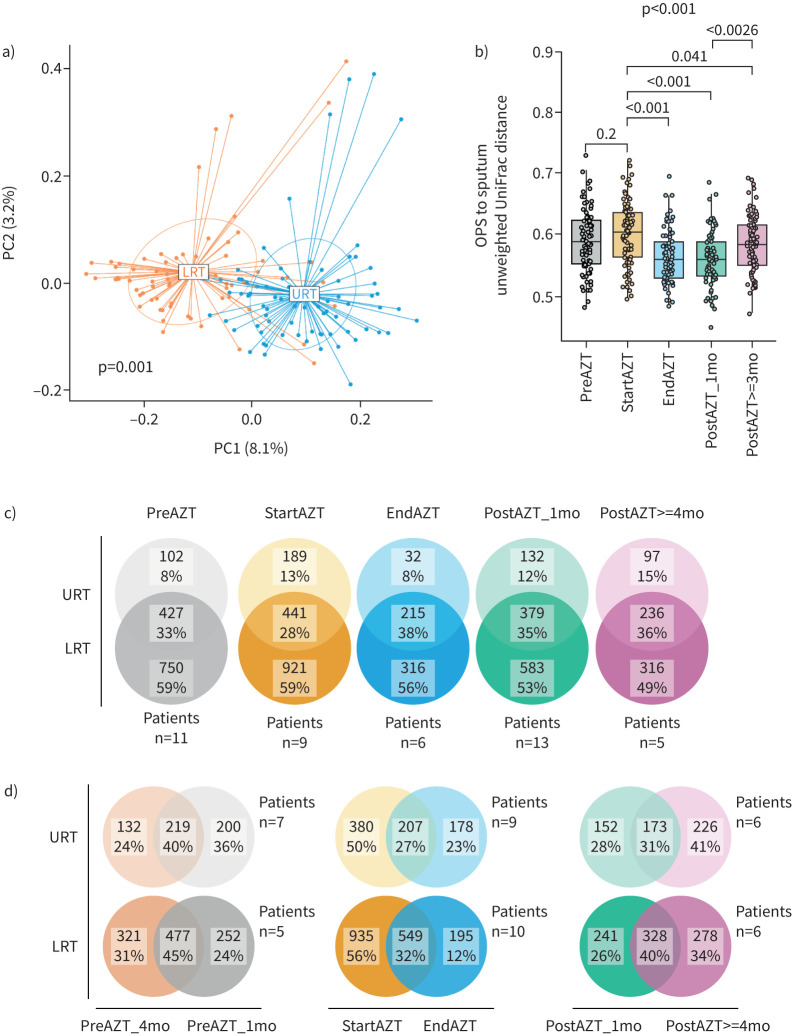
Azithromycin (AZT) treatment reduces dissimilarity between the lower respiratory tract (LRT) and upper respiratory tract (URT) microbiota. a) Principal coordinate analysis (PCoA) based on unweighted UniFrac distance showing an overall difference in bacterial community composition between the URT and LRT. b) Unweighted UniFrac distance calculated for each treatment phase between each oropharyngeal swab (OPS) sample and the centroid obtained from the corresponding set of sputum samples. Middle lines, boxes and whiskers represent the median, interquartile range (IQR) and 1.5×IQR, respectively. Dots represent samples. c) Venn diagrams of the total numbers and proportion of amplicon sequence variants (ASVs) represented in either the LRT or URT alone, or in both sites, showing that the decrease in dissimilarity between the LRT and URT microbiota during treatment was in part driven by a decrease in the proportion of ASVs present in the LRT alone. d) Venn diagrams of the total numbers and proportion of ASVs represented either at the start, at the end or retained over a 3-month time window, showing treatment-induced alterations in the temporal dynamics of the LRT and URT microbiota. mo: months. Significance tested using a) PERMANOVA or b) Kruskal–Wallis with Dunn's *post hoc* test.

**FIGURE 4:**
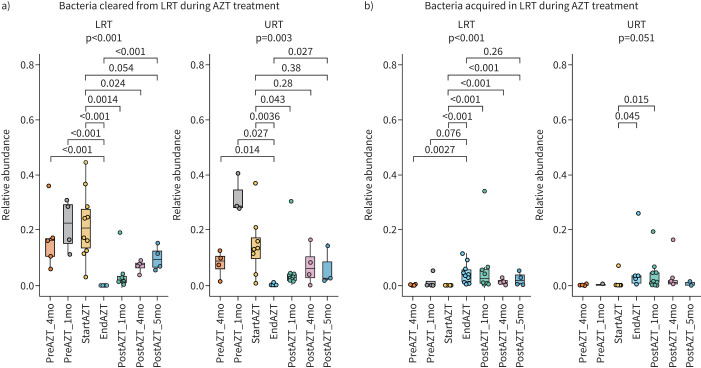
Longitudinal changes in the relative abundance of bacteria a) cleared or b) acquired in the lower respiratory tract (LRT) during azithromycin (AZT) treatment, showing different magnitudes of change in relative abundance between cleared and acquired bacteria, and similar kinetics between the LRT and upper respiratory tract (URT). The relative abundance reported is the proportion of all amplicon sequence variants (ASVs) lost (*i.e.* no reads at the end of treatment) or acquired (*i.e.* no reads at the start of treatment) during treatment, relative to the abundance of total ASVs per sample. Middle lines, boxes and whiskers represent the median, interquartile range (IQR) and 1.5×IQR, respectively. Dots represent samples. mo: months. Kruskal–Wallis with Dunn's *post hoc* test.

Based on unweighted UniFrac distance, bacterial communities in the URT and LRT were distinct (figure 3a). Per-patient comparisons showed greater similarity between URT and LRT samples at the end of AZT treatment, with the effect persisting 1 month after treatment and attenuating thereafter (figure 3b). The impact of treatment was confirmed to be mainly on taxa of relatively low abundance, as indicated by a smaller effect based on weighted UniFrac distance compared with unweighted UniFrac distance (supplementary figure E12; compare with figures 3b and 4b). This treatment-related decrease in dissimilarity between the two sites was also observed at the genus level (supplementary figure E13a).

Divergence of the microbiota between the two sites was driven by ASVs present only in the LRT, more numerous than those present only in the URT or shared between the two sites, in each treatment phase (p<0.05, except at ≥4 months after treatment). The transient decrease in the divergence between the URT and LRT microbiota after AZT was associated with a nonsignificant decrease in the proportion of ASVs represented in the LRT only and a marginal increase in the number of ASVs shared by both sites (figure 3c).

Analysis of samples from IPF patients first receiving placebo allowed quantification of bacterial turnover over a 3-month period in the absence of AZT. We found a total of 40% of ASVs retained in the URT and 45% of ASVs retained in the LRT (figure 3d, left diagrams).

AZT treatment altered the temporal dynamics of the airway microbiota, with fewer ASVs present at the end compared with the start of AZT treatment in the LRT (p<0.001) and URT (p=0.02). In the LRT, the number of newly acquired ASVs was also lower than the number of retained ASVs (p=0.005), in contrast to the observations in patients receiving placebo first (figure 3d, middle diagrams).

However, the bacterial turnover after treatment was increased compared with the placebo-first group, with numbers of newly acquired ASVs exceeding those of cleared ASVs in the LRT (p=0.045) but not reaching significance in the URT (p=0.059) (figure 3d, right diagrams). The observations were confirmed at the genus level (supplementary figure E13b).

We observed that bacteria cleared from the LRT during treatment, which accounted for median (IQR) 20.7% (13.6–27.6%) of the local community at the start of treatment, were already present in this site at 16.5% (11.2–28.6%) relative abundance at least 4 months before treatment started. The effect of treatment on these bacteria was long lasting, with only partial resilience 5 months after the end of treatment. These same bacteria followed similar kinetics in the URT, with a relative abundance of median (IQR) 12.7% (11.2–28.6%) in the 4 months prior to the start of treatment and then decreasing to 0.08% (0.01–0.1%) at the end of treatment (figure 4a).

Bacteria acquired in the LRT during treatment were represented in some cases at low levels during the 4 months prior to the start of treatment. Their local relative abundance was highest at the end of treatment, without reaching the levels of bacteria cleared by the treatment, before decreasing during the 5 months following the end of treatment. These same bacteria were also poorly represented in the URT prior to the start of treatment (median (IQR) 0% (0–0.09%)). At the end of treatment, their local relative abundance had also increased (median (IQR) 2.9% (0.8–3.4%)) and, for some patients, these bacteria remained detectable in the URT for up to 5 months after the end of treatment (figure 4b).

Taken together, these observations indicate marked changes in the spatial distribution and temporal dynamics of the airway microbiota of IPF patients secondary to AZT treatment.

### Changes in composition of the LRT microbiota correlate with ARG carriage

**FIGURE 5:**
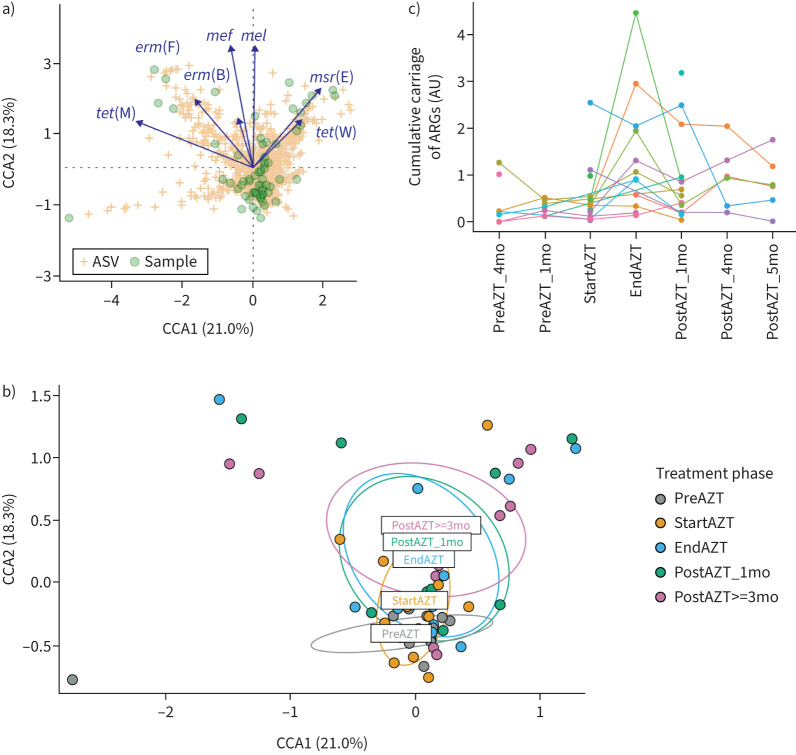
Acquisition of antibiotic resistance genes (ARGs) during azithromycin (AZT) treatment in the lower respiratory tract. a) Canonical correlation analysis (CCA) showing that ARG carriage was limited to a minority of sputum samples and amplicon sequence variants (ASVs) and b) stratification by treatment phase showing that almost all samples with ARGs were taken at the end of AZT treatment or later. c) Within-patient monitoring of the cumulative sum of normalised copy numbers of the full set of ARGs, focusing on patients with more than one sputum sample available, showing the increase in ARG carriage after the start of AZT treatment. AU: arbitrary units; mo: months.

We next investigated whether alterations in the respiratory microbiota during AZT treatment were related to ARG acquisition, focusing on the LRT and previously described target genes [15]. We observed that ARG carriage was limited to a minority of LRT samples and ASVs (figure 5a), and that almost all samples with ARGs were collected at the end of AZT treatment or later (figure 5b). This was confirmed by longitudinal within-patient analysis, which showed a peak in total ARG carriage at the end of AZT treatment, with substantial inter-individual differences in post-treatment kinetics (figure 5c).

To investigate the implications for the LRT microbiota of an increase in ARG carriage, we separated patients with LRT samples available at the start and end of AZT treatment (n=10) into two groups of five patients according to the median change in the cumulative sum of seven pooled ARG (median fold change 3.8) (supplementary figure E14).

**FIGURE 6:**
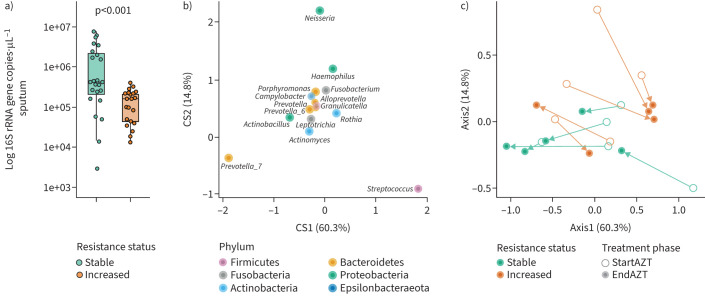
Relationship between the difference in antibiotic resistance gene (ARG) carriage during azithromycin (AZT) treatment, bacterial density and the 15 dominant genera of the lower respiratory tract (LRT) microbiota. a) Quantitative PCR determination of bacterial density showing lower levels in patients with increased resistance. Middle lines, boxes and whiskers represent the median, interquartile range (IQR) and 1.5×IQR, respectively. Dots represent samples. Kruskal–Wallis with Dunn's *post hoc* test. b, c) Double principal coordinate analysis (DPCoA) showing the influence of *Prevotella_7* abundance in the compositional changes occurring during AZT treatment in patients with stable resistance, respectively *Streptococcus* abundance in patients with increased resistance. In b) and c), dots represent different phyla and samples, respectively. In c), the arrows link LRT samples collected from a single patient at the start and end of AZT treatment.

This revealed an association between ARG carriage and bacterial density, with a higher density in patients with stable resistance compared with those with increased resistance during AZT treatment (p<0.001) (figure 6a). However, there was no change in bacterial density between the different treatment phases, whether ARG carriage status was stable or increased (supplementary figure E15).

We observed changes in the composition of the LRT microbiota between the start and end of treatment, depending on resistance status. Specifically, double PCoA (DPCoA) focused on the 15 most abundant genera showed that AZT treatment was associated with a shift in composition influenced by *Prevotella_7* abundance, in all patients with stable resistance and only in one patient with increased resistance. In contrast, the other four patients with increased ARG carriage showed compositional changes mainly driven by *Streptococcus* (figure 6b and c).

Genus abundance analysis during treatment confirmed an increase in *Prevotella_7* and a decrease in *Streptococcus* in patients with stable resistance, and an increase in *Streptococcus* in four out of the five patients with increased resistance (supplementary figure E16a and b). Finally, enrichment of *Prevotella_7* in patients with stable resistance during treatment (p=0.0064) (supplementary figure E16c) and of *Streptococcus* in those with increased resistance (p=0.002) (supplementary figure E16d) was also observed when all samples of these patients were considered, including those taken before the start or after the end of treatment.

Finally, of the 25 genera with the highest number of ASVs lost during treatment, *Treponema_2* and *Fretibacterium* were represented in both patients with stable and increasing resistance status but were absent in a similar ranking of ASVs acquired during treatment (supplementary figure E17a and b).

**FIGURE 7:**
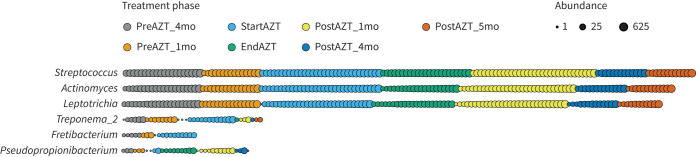
Genera most affected by loss and/or acquisition of amplicon sequence variants in the lower respiratory tract (LRT) during azithromycin (AZT) treatment. Dot plot showing the prevalence (number of dots each representing a sample) and abundance (dot size in log scale) of selected genera during the study phases (colour code). *Streptococcus*, *Actinomyces* and *Leptotrichia* illustrate genera whose abundance and prevalence prior to treatment, and partial replacement during treatment, allowed maintenance throughout the study. *Treponema_2* and *Fretibacterium* on one side, and *Pseudopropionibacterium* on the other, illustrate a decrease, respectively a transient increase, in prevalence linked to treatment. mo: months.

As a result, these genera were significantly less prevalent and less abundant at the end of treatment and in the following months, with no or moderate signs of resilience (for *Fretibacterium* and *Treponema_2*, respectively). On the contrary, *Pseudopropionibacterium* was represented among the ASVs acquired during treatment, with a prevalence still preserved 1 month later (figure 7).

Together, these observations link the resistome to the composition of the LRT microbiota, with a direct correlation with local bacterial density, but without change in lung function during the study period (supplementary figure E18) [21].

## Discussion

This study provides valuable insight on the spatial and temporal distribution of the respiratory microbiota in IPF by comparing the URT and LRT, microbial turnover, and impact of AZT on these dynamics.

Our findings suggest an attenuating effect of AZT on airway ecological disruption but also disturbance in the LRT through a decrease in community diversity and an increase of potential pathogenic community members.

These effects persisted up to 5 months after the end of treatment, possibly due to AZT retention in lung macrophages [27]. No changes in clinical status were observed during the study period [21].

URT and LRT communities in our cohort of IPF patients differed significantly, as in other chronic respiratory diseases [28, 29], with microbial richness in the LRT exceeding that in the URT, which contrasts with the situation in healthy subjects.

Greater decrease in microbial richness in the LRT *versus* URT during AZT treatment, paralleled by a diminishing phylogenetic dissimilarity between the microbiota of the two sites, suggests that treatment decreases airway ecological disturbance. Our observation of no concomitant decrease in bacterial density could imply that the numerous taxa cleared were replaced by a smaller number of taxa already present and becoming more dominant. As increased dominance may alter the bacterial impact on the host, it is often interpreted as being harmful, but little is known about the threshold for detrimental dominance, which depends on the bacteria and the clinical context.

Further evidence of AZT treatment effects was investigated by assessing bacterial temporal dynamics. In the absence of AZT, bacterial turnover was lower in the LRT compared with the URT. Combined with excessive microbial richness in the LRT, this suggests a local persistence of bacteria potentially harmful to the host. AZT treatment had two effects on this microbiota dynamic, namely by eliminating taxa (decrease in richness) and by preventing the acquisition of new taxa. However, these effects vanished progressively during the months following treatment.

The treatment-induced decrease in richness and phylogenetic diversity of the LRT microbiota was linked to a compositional change mainly driven by a mutually exclusive increase in relative abundance of the two predominant genera in IPF lung, *i.e. Streptococcus* (Gram-positive Firmicutes) and *Prevotella* (Gram-negative Bacteroidetes). Enrichment of these genera was consistent with the development of macrolide resistance previously described in cystic fibrosis [30, 31]. The difference we observed with the increase in ARG carriage during AZT treatment when either of these genera predominated suggests that different mechanisms may be present. While *Streptococcus* enrichment was associated with a marked increase in ARG carriage (in four out of four patients), *Prevotella* enrichment was linked to more stable ARG carriage (in five out of six patients). *Prevotella* acquisition of AZT resistance could be due to carriage of genes not listed in our targets, although we included the most likely candidates based on a previous metagenomic study of AZT treatment in severe asthma [15].

A larger sample size will be necessary to strengthen conclusions and to perform in-depth analysis to determine whether carriage of genes involved in tetracycline *versus* macrolide resistance is associated with different bacteria.

Although patients remained clinically stable during the study period, it is difficult to predict the impact of even a relatively transient predominance of *Streptococcus* or *Prevotella* on lung health. Both genera include commensal species that can act as opportunistic pathogens under permissive conditions. Some members of *Streptococcus* have been associated with IPF progression [6]. *Prevotella* are maintained at low levels in the healthy lung, where they establish a subclinical level of inflammation favouring immune surveillance [3]. However, their abundance is increased in IPF where they can become predominant [32]. Furthermore, *Prevotella* are involved in several inflammatory diseases [33], including periodontitis, with a possible impact on the frequency of exacerbations in COPD [34]. Accordingly, pre-clinical evidence suggests that *Prevotella* predominance in the airways promotes pulmonary fibrosis through a mechanism involving interleukin-17B [35]. Decreased microbial diversity in IPF, such as observed with the predominance of *Streptococcus* or *Prevotella*, has previously been associated with increased alveolar concentrations of pro-inflammatory cytokines and profibrotic growth factors [10].

Further studies are required to determine the relationship between alterations of microbiota and gene expression in the airways during AZT treatment.

Our study has several limitations. First, we report an association between AZT and ARG carriage, but separate analysis of microbiota by amplicon sequencing and ARG carriage by qPCR does not allow us to identify bacteria that acquire resistance during treatment. Genome-wide metagenomic analyses would answer this question. Second, the absence of a healthy control group prevented us from assessing whether disruption of the airway microbiota by AZT is specific for IPF. Third, the use of sputum to sample the proximal lower airways in nonsuppurative airway disease such as IPF is associated with a risk of cases being contaminated by saliva. Although we used induction by instillation of hypertonic saline to limit this risk in patients unable to expectorate, the latter sampling method was reported to return differences in the composition of the LRT microbiota compared with spontaneous sputum in a third of patients in the COPD setting [36]. However, the same study found no difference in α diversity between spontaneous and induced sputum. Fourth, we defined bacterial turnover in this cohort of IPF patients by investigating the acquisition or loss of ASVs during the pre-treatment period in patients receiving placebo first. However, some of this variation over time is bound to be related to an artificial inflation of α diversity when measured on the basis of ASVs [37] and randomness related to sequencing depth, rarefaction and sequencing of low-biomass samples [38]. The consistent results of the genus-level analyses suggest that this limitation plays a reasonably restricted role. Finally, our observations are based on the available specimens of 24 well-characterised IPF patients, but the size of the cohort limits the generalisability of our findings which call for larger studies.

Notwithstanding, this study provides the best available evidence on the effect of AZT on the IPF airway microbiota. We report that AZT alters the spatial and temporal dynamics of the airway microbiota in IPF patients with a decline in richness and phylogenetic diversity in the URT and LRT, without impact on bacterial density. The AZT impact is characterised by increased *Prevotella* or *Streptococcus* abundance and persists with partial resilience 5 months after treatment. Also, ARG carriage is increased in half of patients in the presence of *Streptococcus* predominance.

In conclusion, this study provides novel insights into airway ecology disturbances in IPF by longitudinal sampling of the URT and LRT, and expands on previous knowledge from studies in the distal LRT. From a clinical care perspective this approach encourages noninvasive sampling of more than one respiratory tract site.

## Supplementary material

10.1183/23120541.00720-2022.Supp1 **Please note:** supplementary material is not edited by the Editorial Office, and is uploaded as it has been supplied by the author.
Supplementary material 00720-2022.supplement
