# Supplementary material for: Azithromycin alters spatial and temporal dynamics of airway microbiota in idiopathic pulmonary fibrosis
Source: ERJ Open Res. 2023 May 22;9(3):00720-2022. doi: 10.1183/23120541.00720-2022 (PMC10204823; doi:10.1183/23120541.00720-2022)
Supplement: Supplementary file 1 [file 00720-2022.supplement.pdf]

Azithromycin alters spatial and temporal dynamics of airway microbiota in idiopathic pulmonary fibrosis

Authors:

*Pieter-Jan Gijs[a], *Cécile Daccord[a], Eric Bernasconi[a], Martin Brutsche[b], Christian Clarenbach[c], Katrin Hostettler[d], Sabina A. Guler[e], Louis Mercier[a], Niki Ubags[a], +Manuela Funke-Chambour[e] and +Christophe von Garnier[a]

Affiliations

[a] Division of Pulmonology, Department of Medicine, CHUV, Lausanne University Hospital, Lausanne, University of Lausanne, Switzerland
[b] Lung center, Kantonsspital St. Gallen, St. Gallen, Switzerland
[c] Division of Pulmonary Medicine, University Hospital of Zurich, Zurich, Switzerland
[d] Clinics of Respiratory Medicine, University Hospital Basel, Basel, Switzerland
[e] Department of Pulmonary Medicine, Inselspital, Bern University Hospital, Bern, Switzerland

* Shared first authorship
+ Shared last authorship

Corresponding Author:

Niki Ubags
Division of Pulmonology, Department of Medicine, CHUV, Lausanne University Hospital, Lausanne, University of Lausanne, Switzerland
Email : Niki.Ubags@chuv.ch

Supplementary materials and methods

Study population and sample collection

The study was conducted between August 2014 and August 2019; Key inclusion criteria were age over 18 years and a diagnosis of IPF according to current diagnostic guidelines(1). Ethical approval was obtained prior to the start of the study (KEK 002/14), and all patients provided written consent prior to inclusion in the study.

Patients with one or more of the following criteria were excluded: any change in medication or respiratory infection within four weeks prior to inclusion, known allergy or intolerance to macrolide antibiotics, known cardiac arrhythmia, severe renal failure, history of hepatitis, current alcohol or drug abuse, serum bilirubin level of > 50 µmol/L, elevated aspartate transaminase or alanine transaminase by more than three times the upper limit of normal, or QTc prolongation on 12-lead electrocardiogram.

Oropharyngeal swab (OPS) and sputum samples were collected at different time points of the study for characterisation of the microbiota of the upper respiratory tract (URT) and lower respiratory tract (LRT), respectively (**Supplementary Figure E1**). When sputum could not be produced it was induced by inhalation of a 3% sodium chloride solution (details not available if sputum were spontaneous or induced). Samples were frozen at -80°C at each collection centre and then transferred to Lausanne University Hospital for DNA extraction and sequencing.

Bacterial DNA extraction

The reducing agent dithiothreitol (DTT [AppliChem, Darmstad, Germany], final concentration up to 5% for 15 min at room temperature) was used to homogenise the mucus phase of thawed sputum samples. Bacterial DNA from sputum and OPS samples was then extracted using the DNeasy UltraClean microbial kit (Qiagen, Hilden, Germany), modified by pre-incubating with 9000 U Ready-Lyse lysozyme (Epicentre, Hessisch Oldendorf, Germany) for 1 hour at 37°C. Purified DNA was eluted in 30 µl of microbial DNA-free water (Qiagen).

16S rRNA amplicon quantification

The copy numbers of the 16S rRNA gene were determined by qPCR using previously reported primers specific to pan bacteria(2) (see Supplementary Table E00). Amplification was performed using SsoAdvanced Universal SYBR Green Supermix (Bio-Rad, Hercules, CA) on a CFX96 Real-Time detection system (Bio-Rad) with the following cycling parameters: initial 2 min denaturation at 98 °C, followed by 45 cycles of 5 s denaturation at 98 °C, and 60 s annealing/elongation at 61.5 °C. Absolute quantification was performed based on a standard curve obtained with a purified amplicon product.

16S rRNA amplicon sequencing

Bacterial community composition was assessed by Illumina MiSeq sequencing with barcoded primers targeting the V1-V2 region (**Supplementary Table E1**). Amplification was performed using the Accuprime Taq DNA Polymerase High Fidelity kit (Invitrogen, Waltham, MA). Duplicate PCR reactions of 20 µl consisted of 2 µl of 10× Accuprime buffer II, 0.44 µl of each 10 mM barcoded primer F-27 and R-338, 9.03 µl of ultrapure water, 0.09 µl of AccuPrime Taq DNA Polymerase and 8 µl of DNA template with the following cycling parameters: initial 3 min denaturation at 94 °C, followed by 40 cycles of 30 s denaturation at 94 °C, 30 s annealing at 56 °C and 90 s elongation at 72 °C, with a final extension at 72 °C for 5 min. No-template PCR reaction controls (n = 2) were included. Amplicons were quantified using a LabChip GX instrument with the DNA 1 K kit (Perkin Elmer, Waltham, MA), pooled into equimolar amounts and purified using the AMPure XP bead cleaning system (Beckman Coulter, Brea, CA). Libraries were then diluted to 12 pM and spiked with 25% phiX before being loaded onto the Illumina MiSeq platform using pairwise chemistry, generating 250 × 2 read lengths.

Analysis of antibiotic resistance gene carriage

Quantification of carriage of antibiotic resistance genes (ARG) targeting 23S ribosomal RNA methyltransferases (*erm*(B) and *erm*(F)), ATP-binding cassette ribosomal protection protein (*mel* and *msr*[E]), major facilitator superfamily antibiotic efflux pump (*mef*), and tetracycline-resistant ribosomal protection proteins (*tet*[M] and *tet*[W]) was performed on sputum specimens using dye-based (SsoAdvanced Universal SYBR Green, Bio-Rad) or probe-based real-time PCR assays, using primer pairs, probes and conditions previously described(3). To accommodate the limited material available for OPS samples, only *mel* and *tet*(W) gene expression was quantified, using the same qPCR protocol. Absolute quantification was performed based on standard curves obtained with purified amplicon products. The absolute copy number of resistance genes per sample was

normalised to the 16S rRNA gene copy number used as a proxy for bacterial number. To obtain a synthetic picture of ARG carriage per sample, the absolute counts obtained for each individual gene were scaled from 0 to 1 to give equal importance to each gene, and the cumulative counts were reported.

Bioinformatics and statistical analysis

All analyses were performed in R version 4.1.0. Bioinformatics processing, which included demultiplexing, removal of chimeric and short reads, single-base resolution of reads into amplicon sequence variants (ASVs) using the Divisive Amplicon Denoising Algorithm 2 (DADA2) algorithm(4) and taxonomic annotation using the SILVA database(5), was performed using a dedicated pipeline available at https://github.com/chuvpne/dada2-pipeline. Initial abundance filtering of absolute read counts (threshold > 1) was applied in phyloseq 1.38.0, reducing the total number of ASVs detected in all OPS and sputum samples, as well as in controls, from 4,233 to 4,170. Filtering based on ASVs belonging to the Bacteria kingdom further reduced the total number of ASVs from 4,170 to 4,099. We filtered potential contaminants using two complementary methods within decontam 1.14.0 R package(6). The prevalence method, based on the prevalence of ASVs in negative controls versus patient samples, identified 84 contaminants and 4015 non-contaminants (**Supplementary Figure E2a** and **Table E2**). The frequency method is based on the relationship between the relative abundance of ASVs and bacterial density, with the assumption that a negative correlation is characteristic of contaminants. This approach identified 50 contaminants and 4049 non-contaminants (**Supplementary Figure E2b** and **Table E3**). ASV26_Pseudomonas was the only ASV considered as a contaminant by both methods. ASV26_Pseudomonas and ASV6_Cutibacterium showed the strongest contaminant signature (**Supplementary Figure E2a**), as further confirmed by rank analysis of the 15 most abundant ASVs in control samples versus patient samples (**Supplementary Figure E7**). This combined screening identified 133 different ASVs as contaminants that we excluded for downstream analysis.
In addition, 12 OPS samples were excluded based on a sequencing depth not exceeding that in the majority of controls (**Supplementary Figure E5**), despite a bacterial density not similarly low (**Supplementary Figure E4**). Specifically, examination of the sequencing depth per sample (median 3.5×10^4, interquartile range 1.7×10^4 to 5.4×10^4) showed that above the threshold of 10^4, there were 1.4% controls (2 controls, 77 oropharyngeal swabs, 67 sputum samples), whereas below this threshold, the proportion of controls rose to 47.8% (11 controls, 12 oropharyngeal swabs. This latter filtering removed 210 ASVs.
Finally, we performed rarefaction on the remaining 3,756 samples, using the "rarefy_even_depth" command from phyloseq 1.38.0, which set the threshold of reads to 10,055 per sample, and removed ASVs, leaving a total of 3,483 ASVs in 146 samples (**Supplementary Figure E3**).
In downstream analyses, the relative abundance of each ASV was Hellinger transformed(7) using the "decostand" function in vegan 2.6-2. The different alpha diversity measurements were performed using the alpha function in Microbiome 1.16.0. Principal coordinate analysis (PCoA) was used to visualise beta diversity based on unweighted UniFrac distance in vegan 2.6-2 and ggordiplots 0.4.1. Canonical correlation analysis (CCA) was performed using the cca function in vegan 2.6-2 to show the link between variation in respiratory microbiota composition and AR gene carriage. To compare changes in respiratory microbiota between the start and end of AZT treatment in patients with stable vs. increased ARG carriage, we performed a double PCoA, which combines analysis of phylogenetic and abundance data. To this end, we agglomerated the dataset at genus level, retained the 15 most abundant genera, and constructed a phylogenetic tree using the rtree function in ape 5.6-2. DPCoA was then performed using the ordinate function in phyloseq 1.38.0. All analyses were performed in R version 4.1.0.
R scripts are available at https://github.com/CHUVpulmonology/Airway_microbiota-Lung_fibrosis-Azithromycin. The original sequencing data and the starting data for the analyses in R are available at 10.5281/zenodo.7065053.

References

1. Raghu, G, Remy-Jardin M, Myers JL *et al.* Diagnosis of Idiopathic Pulmonary Fibrosis An Official ATS/ERS/JRS/ALAT Clinical Practice Guideline. *Am J Respir Crit Care Med* **198**, 44–68 (2018).
2. Bacchetti De Gregoris T, Aldred N, Clare AS *et al.* Improvement of phylum- and class-specific primers for real-time PCR quantification of bacterial taxa. *J Microbiol Methods* **86**, 351–356 (2011).
3. Taylor SL, Leong LEX, Mobegi FM *et al.* Long-Term Azithromycin Reduces Haemophilus influenzae and Increases Antibiotic Resistance in Severe Asthma. Am J Respir Crit Care Med. **200(**3):309-317 (2019).
4. Callahan BJ, McMurdie PJ, Rosen MJ *et al.* DADA2: High-resolution sample inference from Illumina amplicon data. *Nature Methods 2016 13:7* **13**, 581–583 (2016).
5. Quast C, Pruesse E, Yilmaz P *et al.* The SILVA ribosomal RNA gene database project: improved data processing and web-based tools. *Nucleic Acids Research* **41**, D590 (2013).
6. Davis, NM, Proctor DM, Holmes SP *et al*. Simple statistical identification and removal of contaminant sequences in marker-gene and metagenomics data. *Microbiome* **6**, 1–14 (2018).
7. Legendre P, Gallagher ED. Ecologically meaningful transformations for ordination of species data. *Oecologia* **129**, 271–280 (2001).

Supplementary Figures

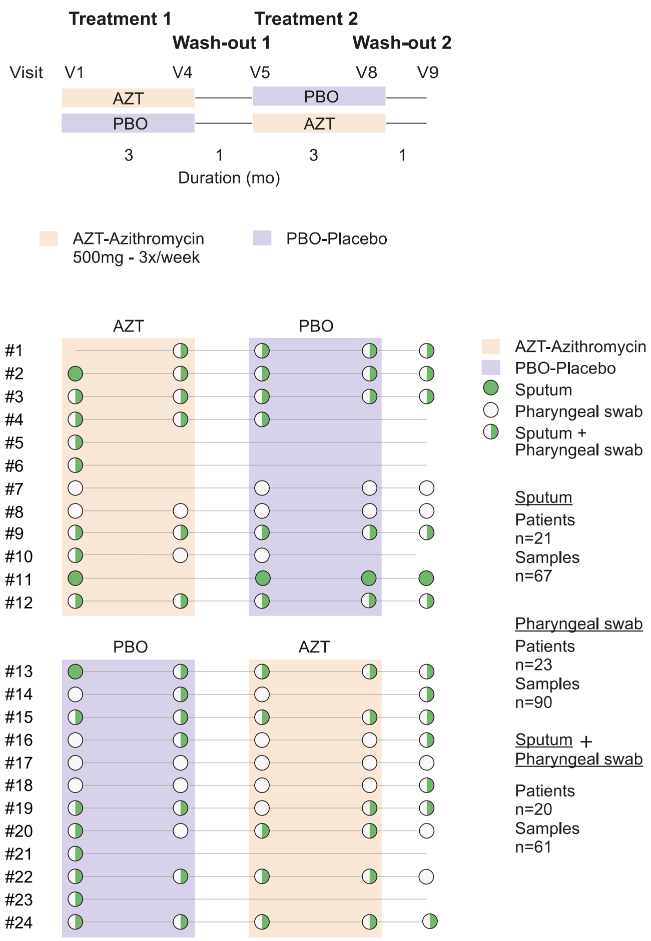

Supplementary Figure E1. Study design and grid of colour-coded collected specimens

a)

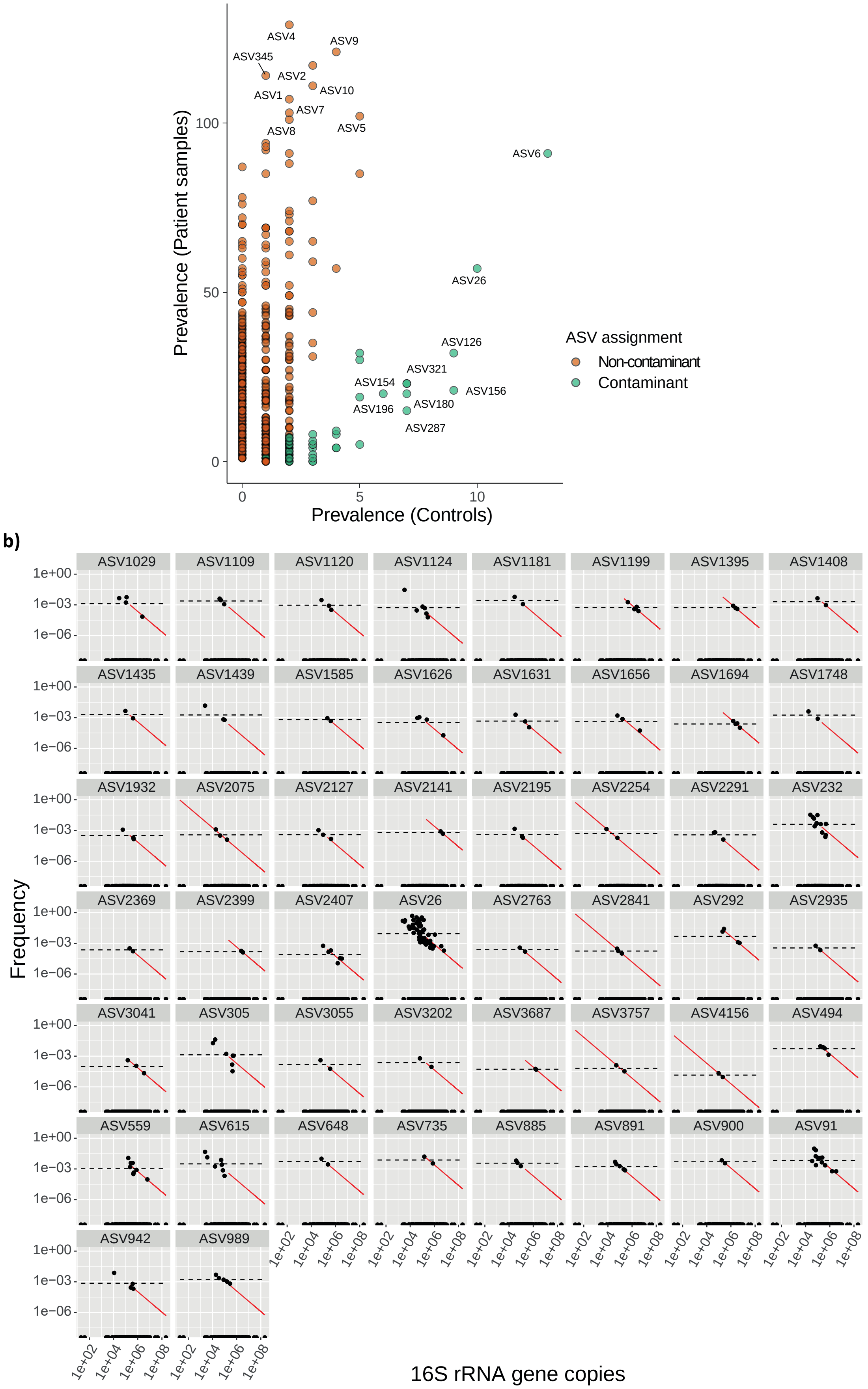

Supplementary Figure E2. Contaminant screening. Contaminants were identified using decontam R package combining the prevalence method based on the prevalence of ASVs in color-coded negative controls versus patient samples (a) and the frequency method based on the relationship between the relative abundance of ASVs (frequency, y-axis) and bacterial density (16S rRNA gene copies, x-axis) (b). The prevalence method and the frequency method identified 50 and 84 contaminants, respectively (see Tables E2 and E3 for the names of the

taxa with the strongest contaminant signatures), with only one ASV (ASV26 - *Pseudomonas* sp.) considered a contaminant in both methods, leading to the filtering out of a total of 133 contaminants.
Abbreviations: ASVs = amplicon sequence variants

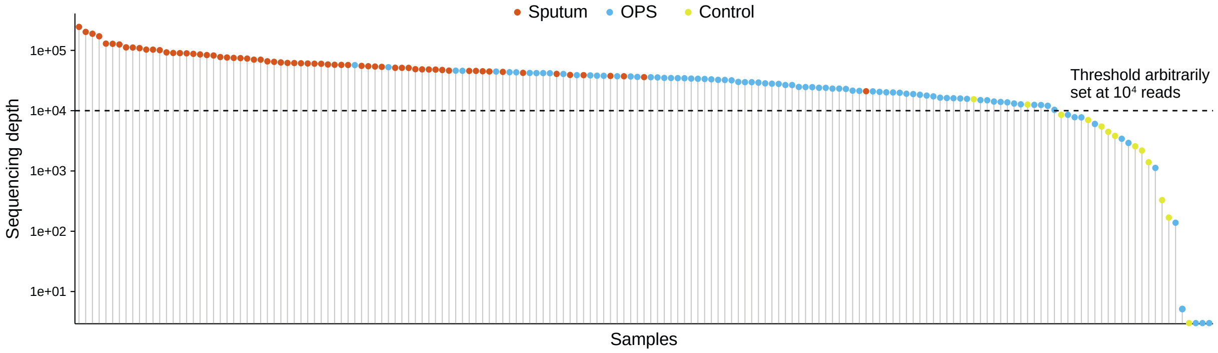

Supplementary Figure E3. Number of reads for each sputum, OPS and control sample. The threshold was arbitrarily set at 1.1 x 10^4 reads. 10 OPS samples were excluded for further analysis.
Abbreviations: OPS = oropharyngeal swab

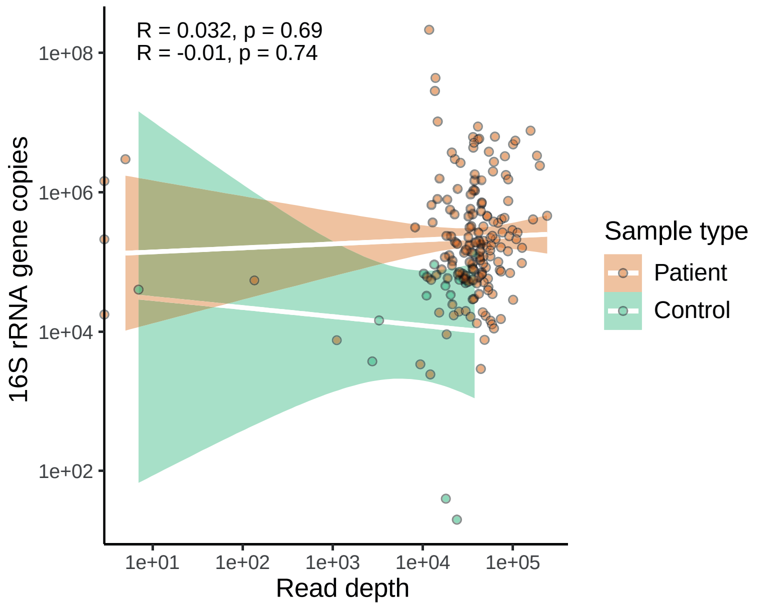

Supplementary Figure E4. Dot plot showing lack of correlation between bacterial density (y-axis) and read depth (x-axis) for patient samples and controls (color coded). Linear models with regression lines and R-squared values and p-values

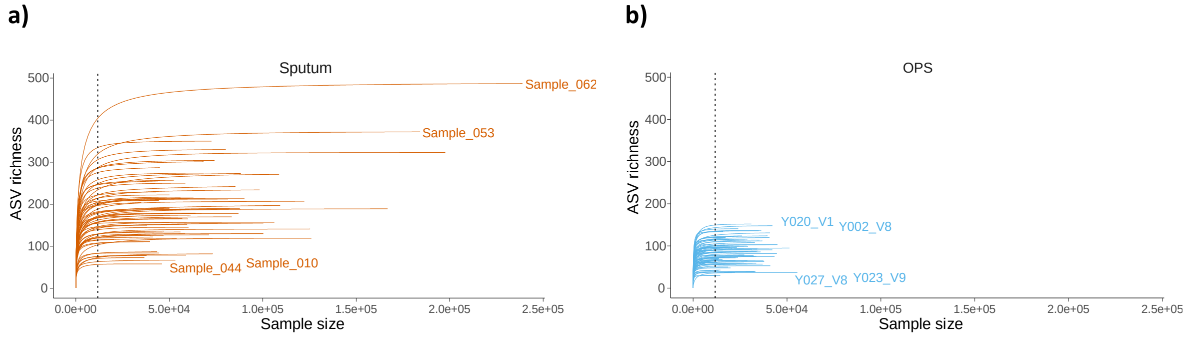

Supplementary Figure E5. Rarefaction curves obtained after abundance-based filtering and contaminant filtering for sputum (*A*) and OPS (*B*) samples, with indication of the 10,055 read depth used in downstream analyses (dashed line).
Abbreviations: OPS = oropharyngeal swab

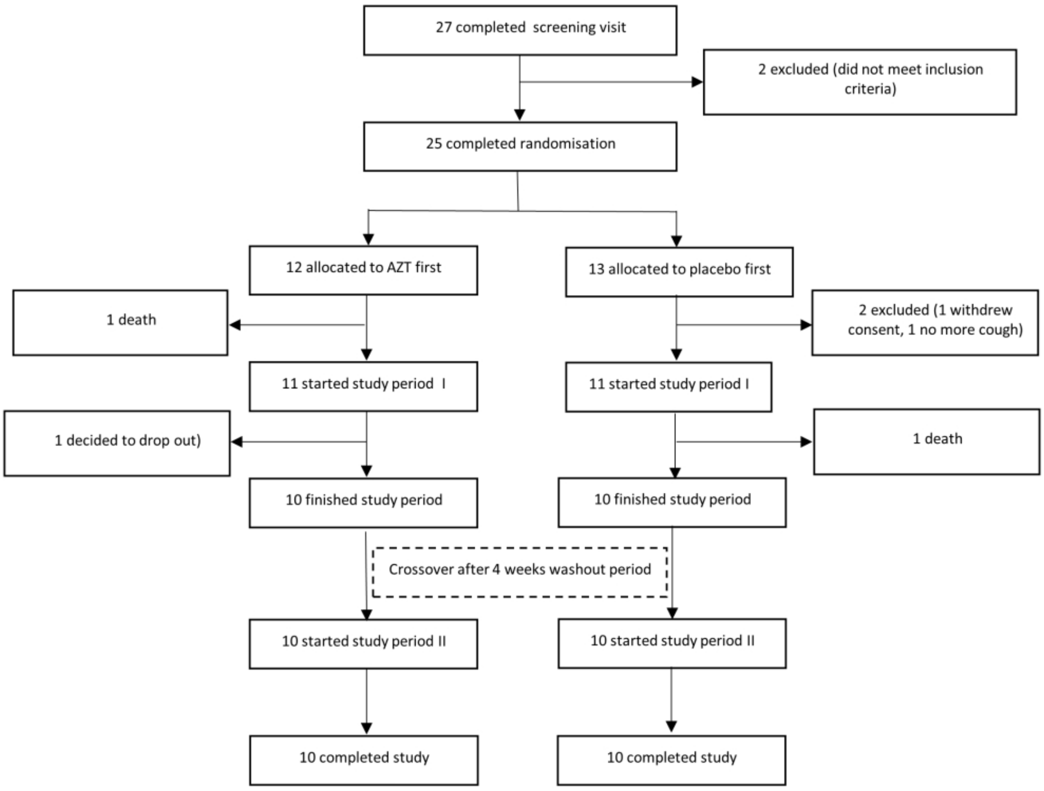

Supplementary Figure E6. CONSORT flow diagram of patients screening, inclusion, and analysis. The "*Azithromycin for the Treatment of Chronic Cough in Idiopathic Pulmonary Fibrosis*" was a prospective, randomized controlled crossover trial, of which the current study is a sub-analysis.

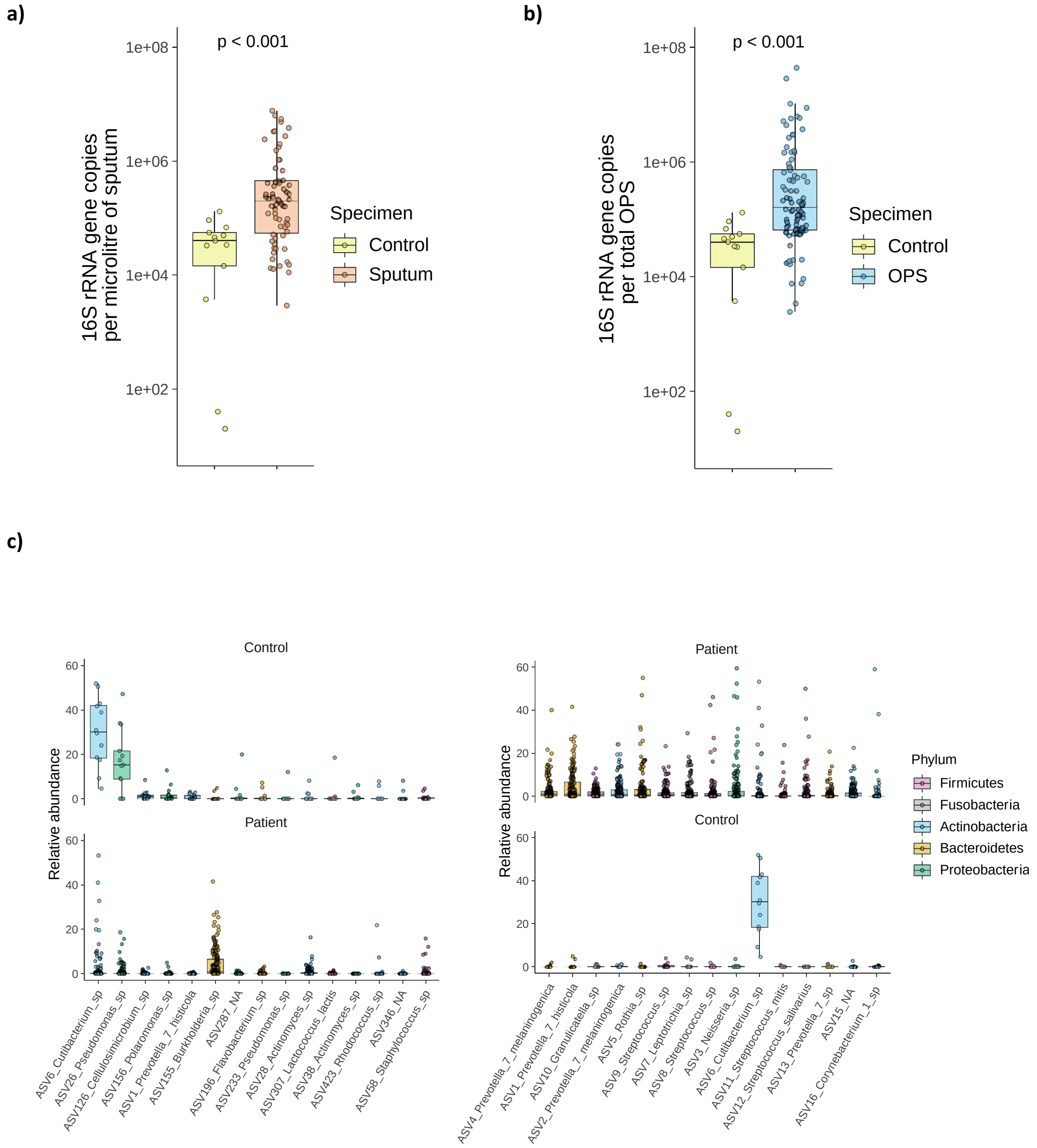

Supplementary Figure E7. Evidence of a distinct bacterial signal between patient samples and negative procedural controls. (*a* and *b*) The bacterial density in sputum (*a*) and OPS (*b*) samples from patients was significantly greater (p<0.001, Wilcoxon rank sum test) than that in negative controls. (*c*) Rank abundance comparison of prominent taxa detected in negative control (left) and patient (sputum combined with OPS, right) samples. For each comparison, the 15 most abundant taxa in each group are displayed in decreasing order of median relative abundance. In all box plots, the middle line, box and whiskers represent the median, interquartile range (IQR) and 1.5 times the IQR, respectively. Dots represent samples.

Abbreviations: OPS = oropharyngeal swab

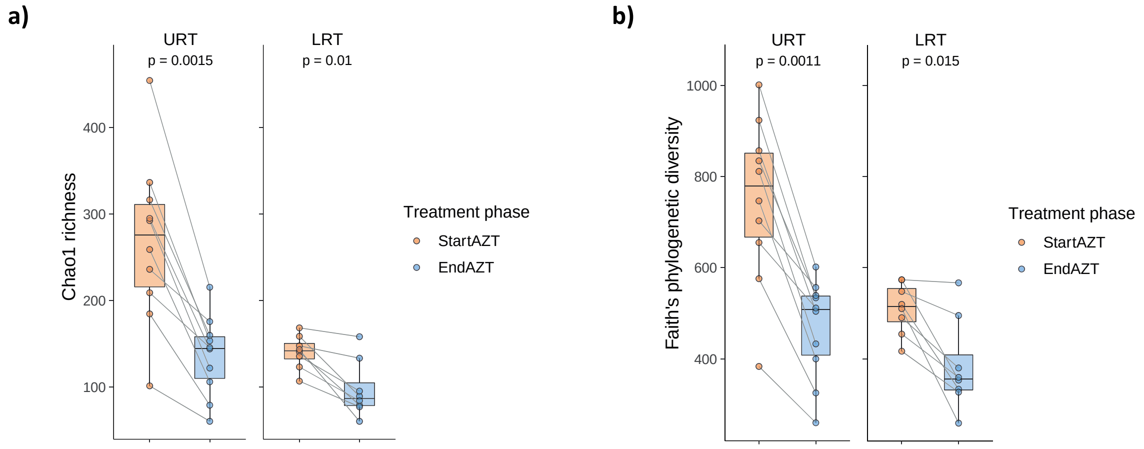

Supplementary Figure E8. Decrease in alpha diversity after AZT treatment. Intra-individual analyses showing a decrease in Chao1 richness (*a*) and Faith's phylogenetic diversity (*b*) in each of the 10 patients for whom sputum sample pairs were available and in each of the 8 patients for whom OPS pairs were available. Middle lines, boxes and whiskers represent the median, interquartile range (IQR) and 1.5 times the IQR, respectively. Dots represent samples and samples from a single patient are paired by a line. Wilcoxon signed-rank test.
Abbreviations: LRT = lower respiratory tract; URT = upper respiratory tract; AZT = azithromycin; OPS = oropharyngeal swab

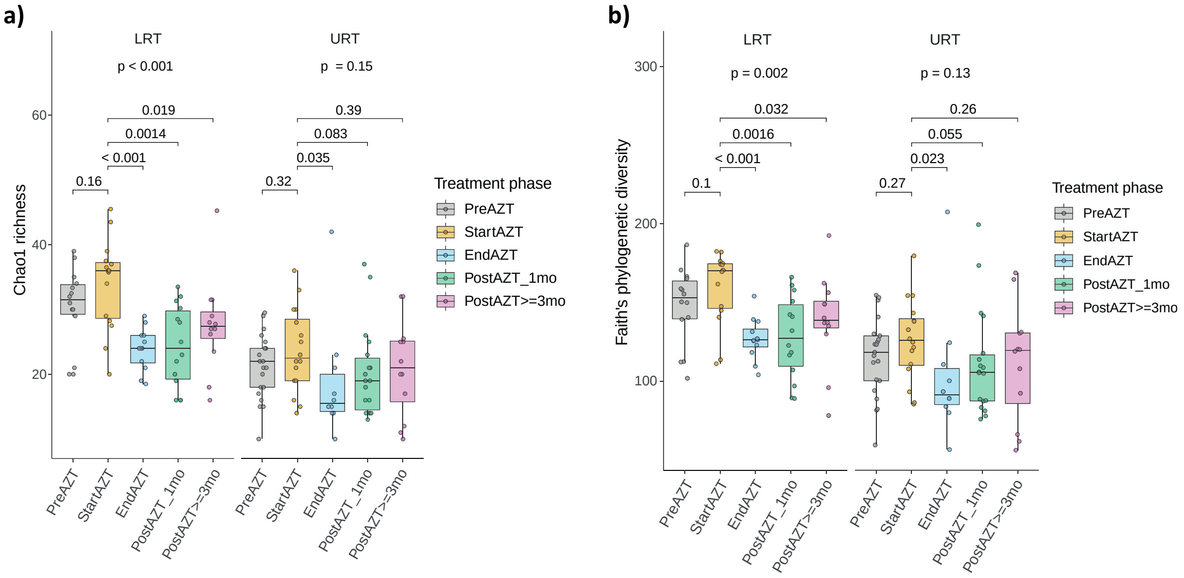

Supplementary Figure E9. Decrease in alpha diversity after AZT treatment, assessed at the genus level. (*a* and *d*) Alpha diversity metrics showing a decrease in Chao1 richness (*a*) and Faith's diversity (*b*) during AZT treatment in LRT and URT. Middle lines, boxes and whiskers represent the median, interquartile range (IQR) and 1.5 times the IQR, respectively. Dots represent samples. Kruskal-Wallis with Dunn's post hoc test.
Abbreviations: AZT = azithromycin; LRT = lower respiratory tract; URT = upper respiratory tract

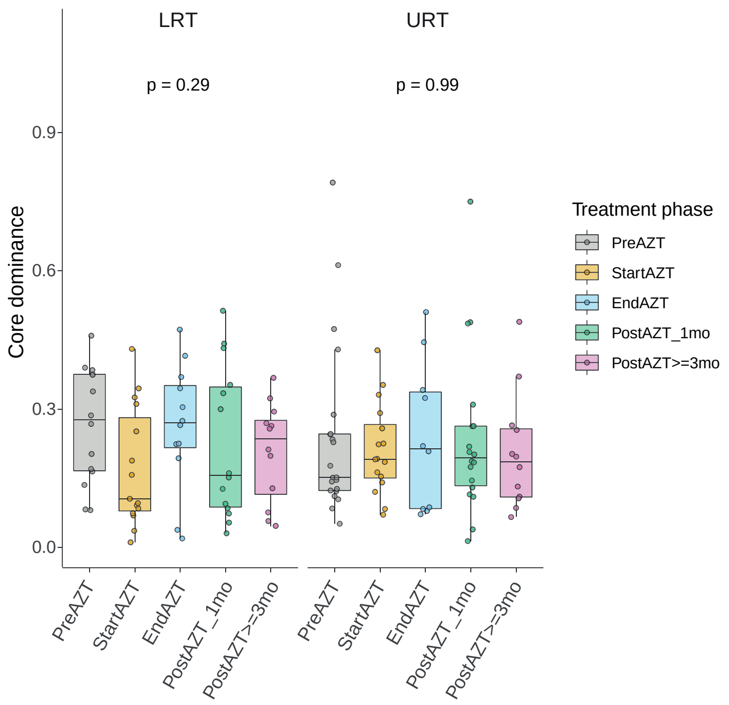

Supplementary Figure E10. Analysis of the dominance of the core community (species exceeding 0.2% relative abundance in more than 50% of the samples) showing no significant difference between the start and end of treatment in LRT or URT. Middle lines, boxes and whiskers represent the median, interquartile range (IQR) and 1.5 times the IQR, respectively. Dots represent samples. Kruskal-Wallis test.
Abbreviations: AZT = azithromycin; LRT = lower respiratory tract; URT = upper respiratory tract

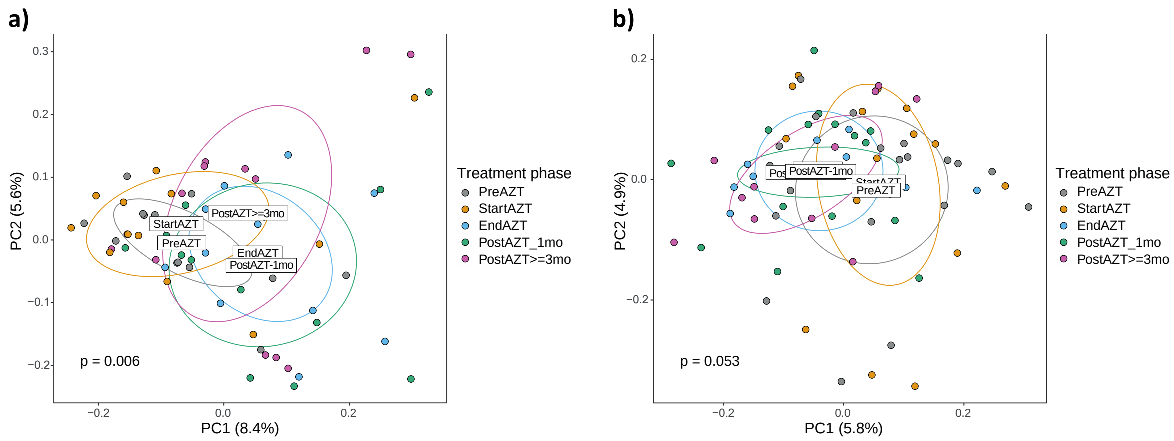

Supplementary Figure E11 (*a* and *b*) PCoA of bacterial communities based on unweighted UniFrac distance showing that the community composition of airway bacteria was distinct between specimens collected before or at the start of AZT treatment, compared to those collected at the end of treatment or later, in LRT (*a*). There was no significant difference in URT (*b*). In PCoA labels indicate the position of the centroid of the corresponding group, the label "EndAZT" is hidden by the label "PostAZT_1mo". PERMANOVA.
Abbreviations: PCoA = principal coordinate analysis; AZT = azithromycin; LRT = lower respiratory tract; URT = upper respiratory tract

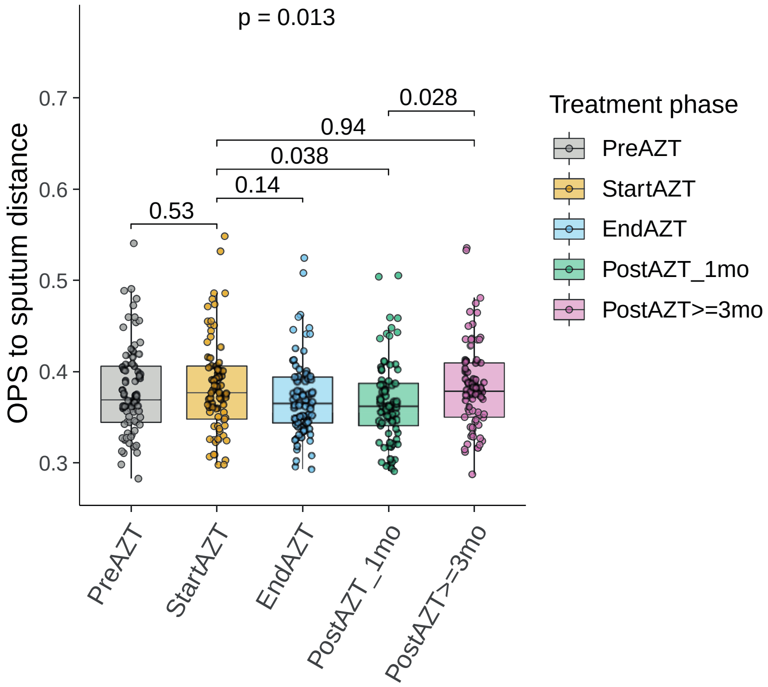

Supplementary Figure E12. AZT treatment transiently reduces dissimilarity between LRT and URT microbiota based on weighted UniFrac distance. For each treatment phase, weighted UniFrac distance was calculated between each OPS sample and the centroid obtained from the corresponding set of sputum samples. The weaker impact of AZT treatment on dissimilarity between LRT and URT microbiota when based on weighted UniFrac distance compared to unweighted UniFrac distance indicates that relatively low abundance taxa are mainly affected. Middle lines, boxes and whiskers represent the median, interquartile range (IQR) and 1.5 times the IQR, respectively. Dots represent samples. Kruskal-Wallis with Dunn's post hoc test.

Abbreviations: AZT = azithromycin; OPS = oropharyngeal swab; LRT = lower respiratory tract; URT = upper respiratory tract

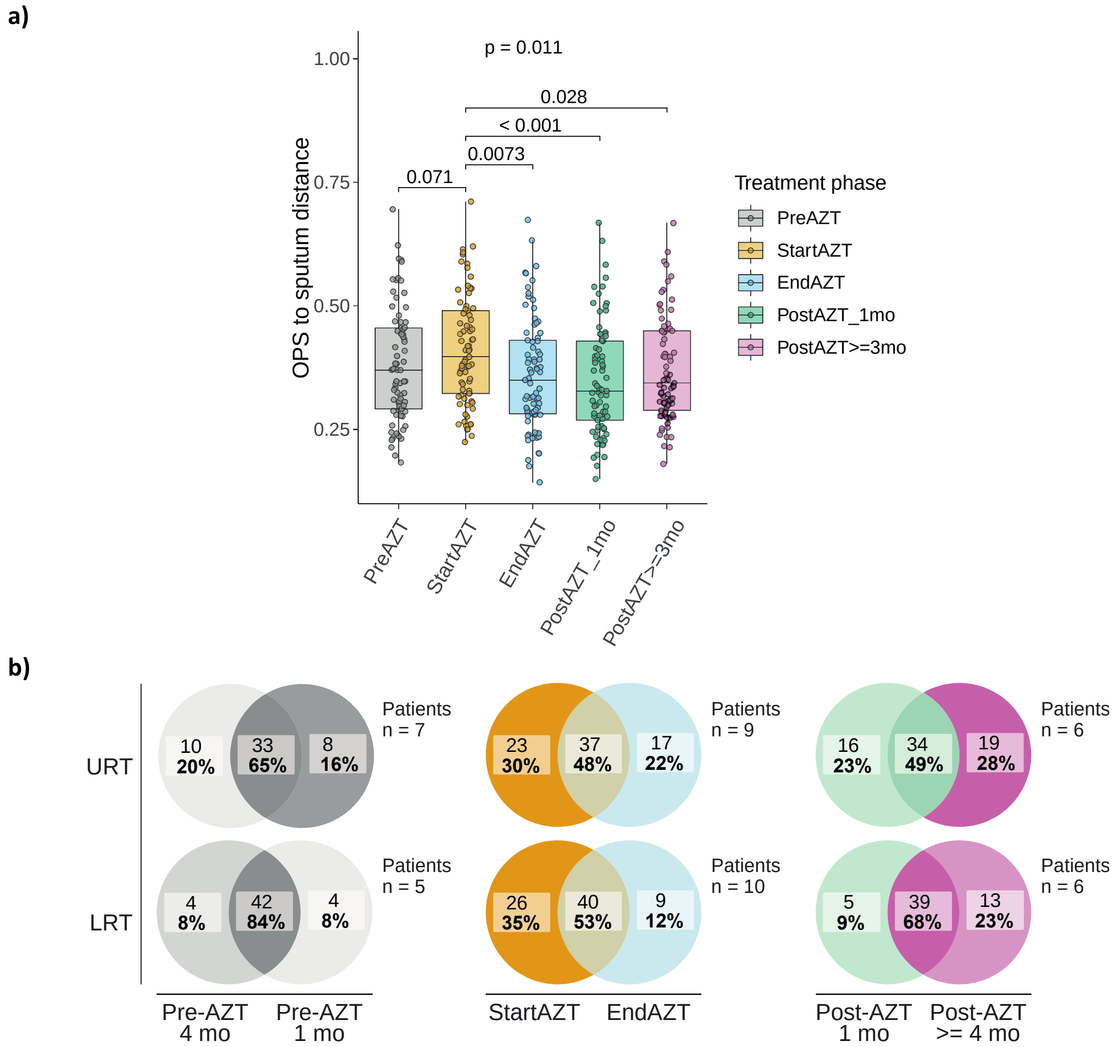

Supplementary Figure E13. AZT treatment reduces dissimilarity between LRT and URT microbiota at genus level. (*a*) Unweighted UniFrac distance calculated for each treatment phase between each OPS sample and the centroid obtained from the corresponding set of sputum samples. Middle lines, boxes and whiskers represent the median, interquartile range (IQR) and 1.5 times the IQR, respectively. Dots represent samples. Kruskal-Wallis with Dunn's post hoc test. (*b*) Venn diagrams of the total numbers and proportions of genera represented either at the start, at the end or retained over a 3-month time window, showing treatment-induced alterations in the temporal dynamics of the LRT and URT microbiota.

Abbreviations: AZT = azithromycin; LRT = lower respiratory tract; URT = upper respiratory tract; OPS = oropharyngeal swab

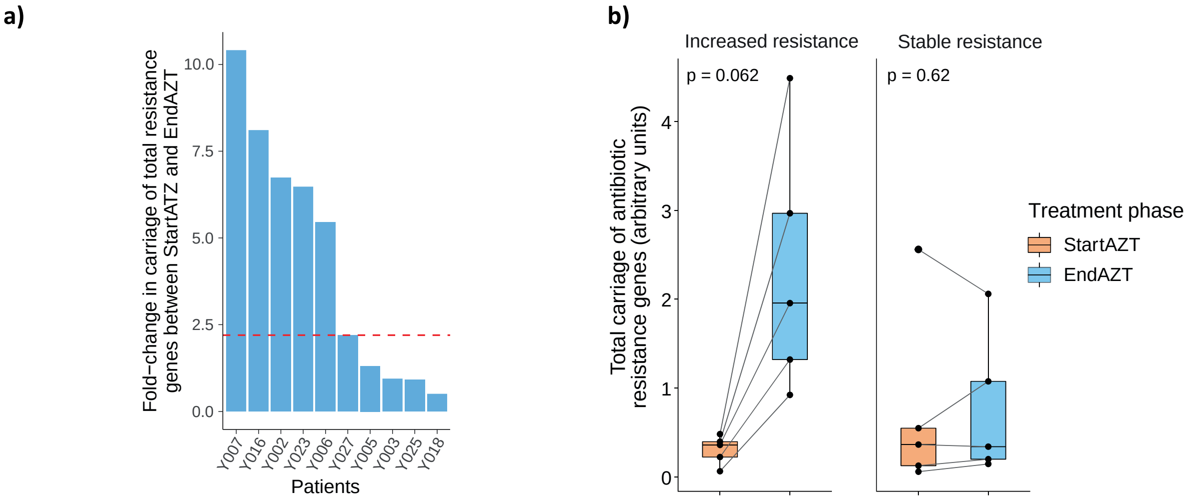

Supplementary Figure E14. Patient-specific fold-change in the cumulative sum of seven pooled ARG in the LRT during AZT treatment. (*a*) Separation into two groups of patients according to the median fold-change (3.8; red dotted line; ranges 5.5-10.4 and 0.51-2.2 for the groups of patients considered to have shown increased resistance, respectively stable resistance, during treatment). (*b*) Kinetics of ARG carriage in the two groups of patients with increased or stable resistance during AZT treatment. Middle lines, boxes and whiskers represent the median, interquartile range (IQR) and 1.5 times the IQR, respectively. Dots represent samples and samples from a single patient are paired by a line. Wilcoxon signed-rank test.

Abbreviations: ARG = antibiotic resistance genes; LRT = lower respiratory tract; AZT = azithromycin

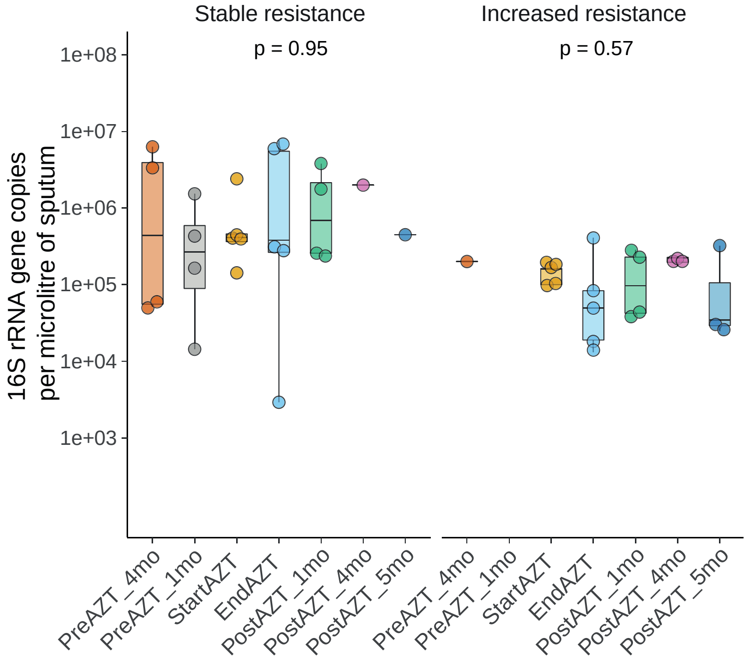

Supplementary Figure E15. Bacterial density evolution between treatment phases in LRT. There was no change in bacterial density between treatment phases, regardless of ARG carriage. Middle lines, boxes and whiskers represent the median, interquartile range (IQR) and 1.5 times the IQR, respectively. Dots represent samples. Kruskal-Wallis test.

Abbreviations: LRT = lower respiratory tract; ARG = antibiotic resistance genes

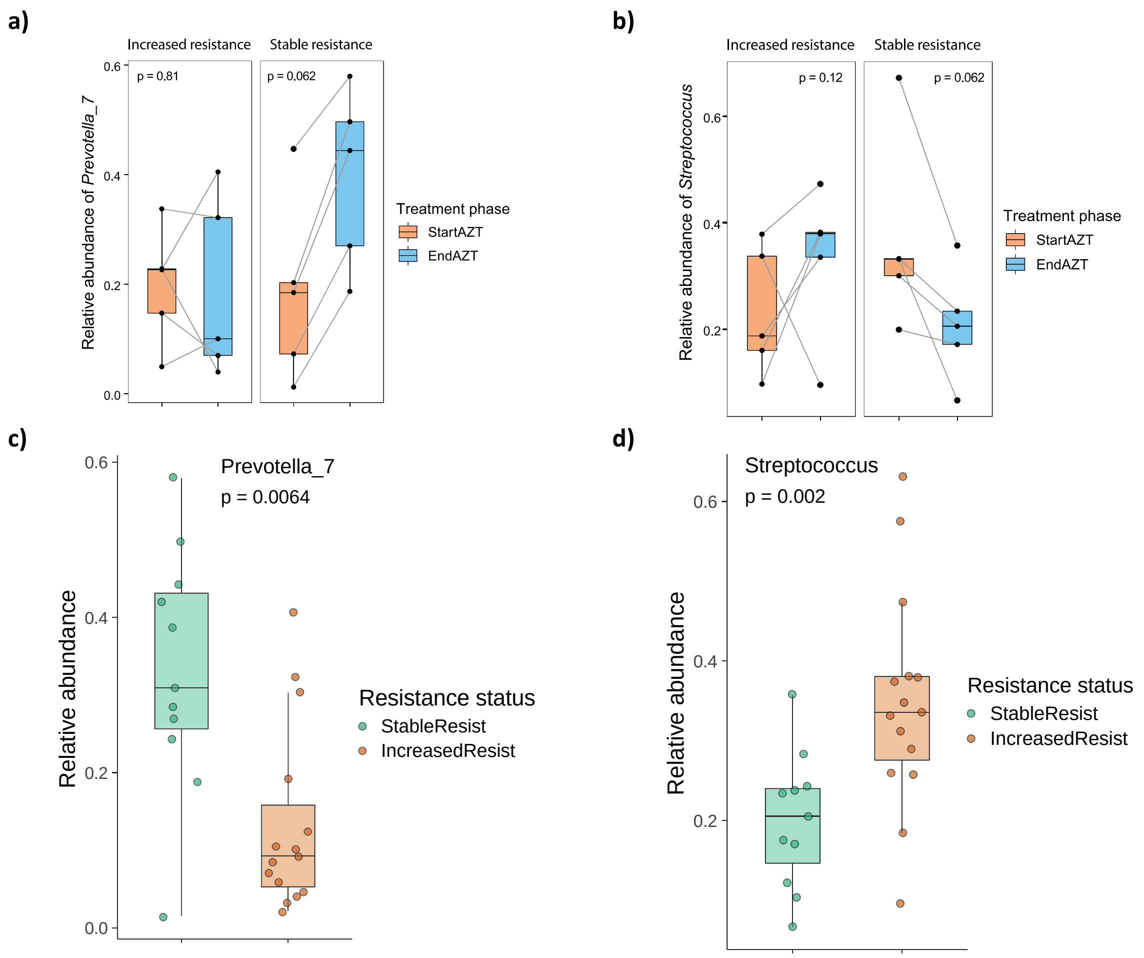

Supplementary Figure E16. Changes in the composition of the LRT microbiota in relation to ARG carriage during AZT treatment. (*a*) Increase in the relative abundance of the genus *Prevotella_7* in all five patients with stable ARG carriage and more disparate changes in patients with increased ARG carriage. (*b*) Decrease in the relative abundance of the genus *Streptococcus* in all five patients with stable ARG carriage and more disparate changes in patients with increased ARG carriage. (*c*) Relative abundance of *Prevotella_7* showing higher levels in patients with stable resistance. (*d*) Relative abundance of *Streptococcus* showing higher levels in patients with increased resistance. Middle lines, boxes and whiskers represent the median, interquartile range (IQR) and 1.5 times the IQR, respectively. Dots represent samples. Samples from a single patient are paired by a line. Significance tested using Wilcoxon signed-rank test (*a* and *b*) or Wilcoxon rank sum test (*c* and *d*). *Abbreviations*: LRT = lower respiratory tract; ARG = antibiotic resistance genes; AZT = azithromycin

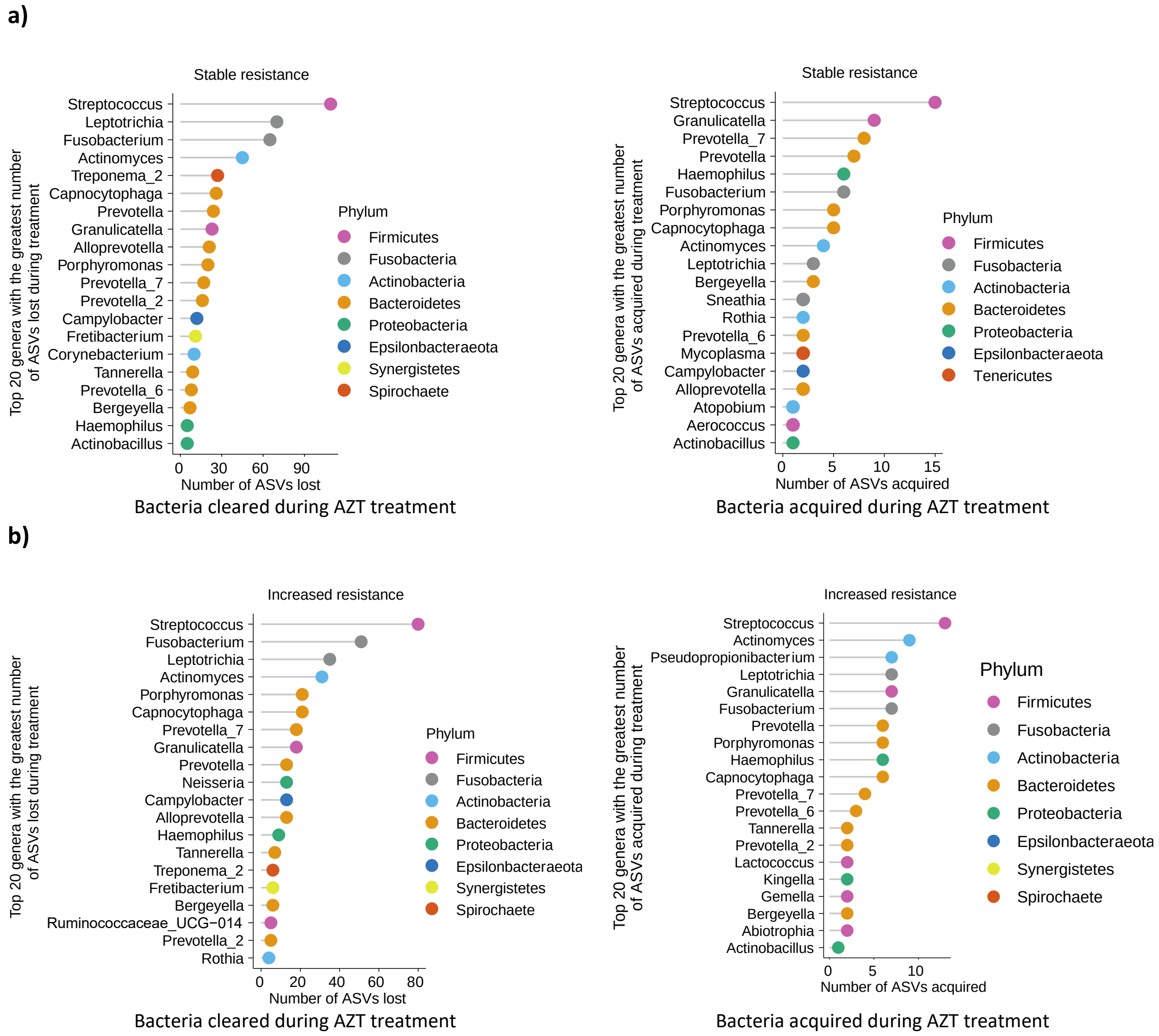

Supplementary figure E17. Genera most affected by loss and/or acquisition of ASVs in sputum during AZT treatment. (*a* and *b*) Lollipop chart showing for patients with stable (a) or increased (b) ARG carriage the number of ASVs lost (left panels) or acquired (right panels) during treatment, classified by genus. Regardless of resistance status, *Streptococcus*, *Leptotrichia*, *Fusobacterium* and *Actinomyces* were the genera with the highest number of ASVs lost during treatment and were also represented among the lower numbers of ASVs acquired during treatment, suggesting partial replacement

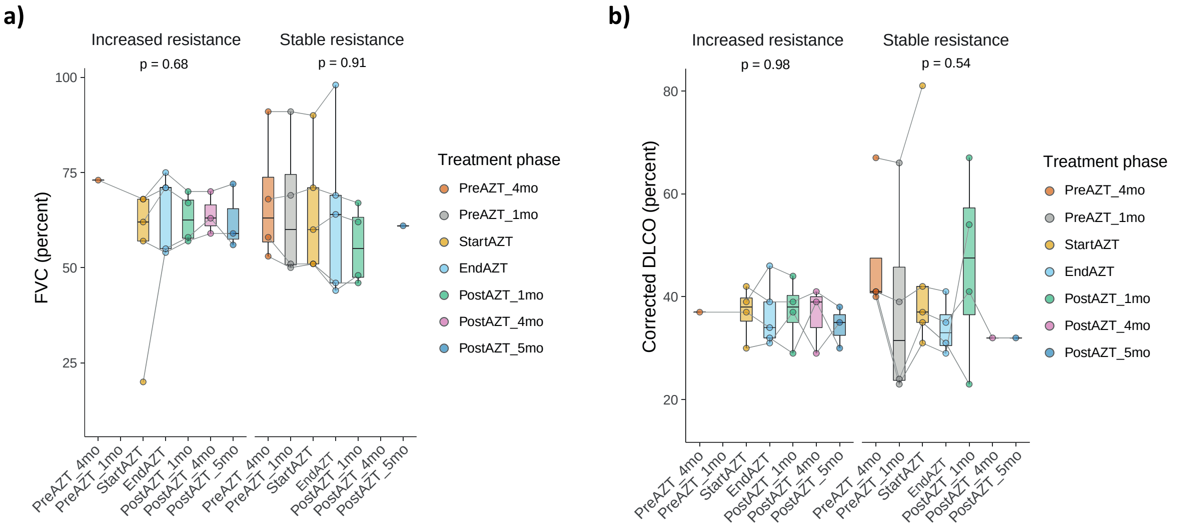

Supplementary Figure E18. Links between AZT treatment-related carriage of ARG in the LRT microbiota and lung function curves. (*a* and *b*) Analysis of LRT samples from patients with available ARG carriage status (n = 10) showing no consistent variations between patients in FVC (*a*) and DLCOc (*b*) during the different phases of treatment, independently of AR gene carriage. Middle lines, boxes and whiskers represent the median, interquartile range (IQR) and 1.5 times the IQR, respectively. Dots represent samples and samples from a single patient are paired by a line. Kruskal-Wallis test.

Abbreviations: AZT = azithromycin; ARG = antibiotic resistance genes; LRT = lower respiratory tract; FVC = forced vital capacity; DLCOc = corrected diffusing capacity of the lung for carbon monoxide

Supplementary Tables

Table E1. Oligonucleotide primers for 16S ribosomal RNA gene analysis.

Name	Method	Sequence (5'-3')
27F	Illumina MiSeq	**AATGATACGGCGACCACCGAGATCTACAC***TATGGTAATTCC*AGMGTTYGATYMTGGCTCAG
338R	Illumina MiSeq	**CAAGCAGAAGACGGCATACGAGAT**NNNNNNNNNNNN*AGTCAGTCAGAA*GCTGCCTCCCGTAGGAGT
926F	qPCR	AAACTCAAAKGAATTGACGG
1062R	qPCR	CTCACRRCACGAGCTGAC

Note: 27F and 338R Illumina sequencing primers allow to amplify the V1-V2 hypervariable region of the 16S rRNA gene. Primers 926F and 1062R target conserved sequences flanking the V6 hypervariable region of the 16S rRNA gene (Bacchetti de Gregoris, doi: 10.1016/j.mimet.2011.06.010). Boldface indicates Illumina adapter sequences, italicized characters indicate linkers, and underlined characters indicate sequences annealing to the target gene. The sequence NNNNNNNNNNNN represents the sample-specific molecular identification barcode.

Table E2. Prevalence-based contaminants

ASV[a]	Phylum[b]	Family[b]	Genus[b]	Species[b]
ASV6	Actinobacteria	Propionibacteriaceae	Cutibacterium	
ASV26	Proteobacteria	Pseudomonadaceae	Pseudomonas	
ASV156	Proteobacteria	Burkholderiaceae	Polaromonas	
ASV126	Actinobacteria	Promicromonosporace	Cellulosimicrobium	
ASV287	Actinobacteria	Nocardiaceae		
ASV321	Proteobacteria	Enterobacteriaceae		
ASV180	Actinobacteria	Propionibacteriaceae	Cutibacterium	granulosum
ASV154	Actinobacteria	Corynebacteriaceae	Lawsonella	
ASV196	Bacteroidetes	Flavobacteriaceae	Flavobacterium	
ASV326	Firmicutes	Staphylococcaceae	Staphylococcus	
ASV16	Actinobacteria	Corynebacteriaceae	Corynebacterium_1	
ASV307	Firmicutes	Streptococcaceae	Lactococcus	lactis
ASV58	Firmicutes	Staphylococcaceae	Staphylococcus	
ASV155	Proteobacteria	Burkholderiaceae	Burkholderia-Caballeronia-Paraburkholderia	
ASV661	Proteobacteria	Burkholderiaceae		

Note: [a]Numbers and corresponding [b]taxonomic assignment of ASVs identified as contaminants by the prevalence-based method implemented in decontam R package (see Supplementary Methods)

Table E3. Frequency-based contaminants

ASV[a]	Phylum[b]	Family[b]	Genus[b]	Species[b]
ASV26	Proteobacteria	Pseudomonadaceae	Pseudomonas	
ASV891	Firmicutes	Streptococcaceae	Streptococcus	
ASV2369	Firmicutes	Streptococcaceae	Streptococcus	
ASV2399	Bacteroidetes	Prevotellaceae	Prevotella 6	
ASV3041	Actinobacteria	Bifidobacteriaceae	Bifidobacterium	dentium
ASV1932	Bacteroidetes	Flavobacteriaceae	Capnocytophaga	
ASV1181	Fusobacteria	Fusobacteriaceae	Fusobacterium	
ASV292	Firmicutes	Streptococcaceae	Streptococcus	
ASV648	Firmicutes	Streptococcaceae	Streptococcus	
ASV1395	Fusobacteria	Leptotrichiaceae	Leptotrichia	
ASV900	Fusobacteria	Leptotrichiaceae	Leptotrichia	
ASV1439	Actinobacteria	Corynebacteriaceae		
ASV3055	Patescibacteria	Saccharimonadaceae		
ASV4156	Firmicutes	Streptococcaceae	Streptococcus	
ASV1585	Bacteroidetes	Flavobacteriaceae	Capnocytophaga	

Note: [a]Numbers and corresponding [b]taxonomic assignment of ASVs identified as contaminants by the frequency-based method implemented in decontam R package (see Supplementary Methods)
